# Implantable device actuated by manual button clicks for noninvasive self‐drug administration

**DOI:** 10.1002/btm2.10320

**Published:** 2022-04-05

**Authors:** Cho Rim Kim, Yong Chan Cho, Seung Ho Lee, Jae Hoon Han, Min Ji Kim, Han Bi Ji, Se‐Na Kim, Chang Hee Min, Byung Ho Shin, Cheol Lee, Young Min Cho, Young Bin Choy

**Affiliations:** ^1^ Interdisciplinary Program in Bioengineering, College of Engineering Seoul National University Seoul South Korea; ^2^ Institute of Medical and Biological Engineering, Medical Research Center Seoul National University Seoul South Korea; ^3^ Department of Biomedical Engineering Seoul National University College of Medicine Seoul South Korea; ^4^ Department of Pathology Seoul National University College of Medicine Seoul South Korea; ^5^ Department of Internal Medicine Seoul National University College of Medicine Seoul South Korea; ^6^ Department of Translational Medicine, College of Medicine Seoul National University Seoul South Korea

**Keywords:** implantable device, metabolic disorders, on‐demand drug delivery, self‐injectable therapy

## Abstract

Self‐injectable therapy has several advantages in the treatment of metabolic disorders. However, frequent injections with needles impair patient compliance and medication adherence. Therefore, we develop a fully implantable device capable of on‐demand administration of self‐injection drugs via noninvasive manual button clicks on the outer skin. The device is designed to infuse the drug only at the moment of click actuation, which allows for an accurate and reproducible drug infusion, and also prevents unwanted drug leakage. Using a mechanical means of drug infusion, this implantable device does not contain any electronic compartments or batteries, making it compact, and semi‐permanent. When tested in animals, the device can achieve subcutaneous injection‐like pharmacokinetic and pharmacodynamic effects for self‐injection drugs such as exenatide, insulin, and glucagon.

## 1 INTRODUCTION

Injectable medications have transformed our ability to effectively treat chronic metabolic disorders, such as diabetes mellitus and obesity, which are significant public health problems worldwide.^1^, ^2^, ^3^ To treat chronic disorders, self‐injection has been reported to increase the flexibility of the time and place of drug administration, reduce the cost to both the patient and healthcare system, and reduce the burden to caregivers.^4^, ^5^ Given these advantages, many drugs, mostly macromolecular drugs, have been widely used for self‐injections in clinical settings.^6^, ^7^

In practice, however, many patients have difficulty learning and performing self‐injection.^8^, ^9^, ^10^, ^11^ Fear of injection is widespread, affecting more than 80% of children and adults.^12^, ^13^ About 20%–30% of patients are known to suffer from extreme fear of needles,^12^ which is reported to affect more than one third of diabetic patients needing multiple daily injections of insulin.^14^ Other psychological issues related to self‐injection, such as anxiety, lack of confidence, and embarrassment, can also negatively influence adherence to a prescribed medication schedule.^5^, ^15^, ^16^ More than half of patients are reported to intentionally skip administration of self‐injected drugs.^14^, ^17^ Lack of *adherence to a prescribed* regimen is a *serious* problem in clinical settings, leading to significant worsening of disease, increased medical health care costs, and even death.^18^

In the light of these findings, it is clear that an implantable drug delivery device could be a promising approach to the delivery of self‐injection drugs. With one‐time implantation, the device inside the body can administer an accurate dose of drugs on‐demand and, more importantly, without needles.^19^, ^20^, ^21^, ^22^ In order to achieve this aim, however, many implantable devices need to use electronic circuits and batteries, and are thus relatively large for implants. When the battery runs out, surgery for device replacement is inevitable, a situation of which further reduces patient acceptance of implantable drug delivery devices.^19^, ^20^

To address these problems, we developed an implantable drug delivery device actuated via mechanical means. Taking into account ease of use, we developed a fully implantable button click device (BCD) in this study, which contains a button to be manually clicked on the outer skin for device actuation for the administration of a bolus of a drug. The BCD described here is rod‐shaped for easy implantation. The button in the BCD is located at one end of the rod‐shaped body, and the body is small enough to be gripped with the fingers. Therefore, the button can be found through the skin over the implanted BCD, and can be pressed with the fingers, noninvasively. The click actuation is created when the button is pressed to a certain level, which generates a constant pushing force. Thus, at the time of clicking, a specific amount of a drug can be accurately infused, and the drug dose can easily be controlled by varying the number of button clicks. The BCD is also equipped with a drug container and refill port. The refill port can be found and accessed from outside the body after implantation, thereby allowing multiple drug replenishments and semi‐permanent use without device replacement surgery.

We evaluated a fully implantable BCD for noninvasive delivery of a self‐injection drug. We used exenatide, a short‐acting glucagon‐like peptide‐1 (GLP‐1) receptor agonist, which is prescribed for diabetic patients, to be self‐injected once or twice per day.^23^, ^24^ The BCD was implanted and delivered exenatide in animal models of obesity and Type 2 diabetes, and the results were comparable with those of animals treated with conventional needled injections. In order to broaden the range of applications, the BCD was also assessed with other self‐injection drugs, such as prandial insulin and glucagon, and the pharmacodynamic (PD) profile—in this case the plasma glucose level—was examined and compared with that of the animals treated with needled injections.

## 2 MATERIALS AND METHODS

### 2.1 Materials

Veroclear and SUP706 were purchased from Stratasys (Rehovot) as 3D‐printing and supporting materials, respectively. The stainless‐steel springs were purchased from Tohatsu. Polyurethane films, 100 μm in thickness, and Teflon molds, were obtained from CY International and 3DMD, respectively. Rubber piston stoppers were obtained by disassembly from a 0.3 ml BD ultra‐fine insulin syringe. Medical epoxy (Epo‐tek 301) was purchased from Epoxy Technology. Phosphate‐buffered saline (PBS) and formalin were obtained from Thermo Fisher Scientific. The check valves were purchased from Minivalve. Trifluoracetic acid (TFA) was purchased from Sigma‐Aldrich. Exenatide (MW = 4187 Da) was purchased from Cosmogenetech. Both insulin and glucagon were obtained from NovoNordisk.

### 2.2 BCD fabrication

The constituent units of the BCD were created by 3D CAD using Autodesk Fusion 360 (Autodesk), and then fabricated with a 3D printer (Objet 30 Pro, Stratasys Rehovot). The printed units were assembled using medical epoxy, as depicted in Figure S1. To prepare the piston, a 9.4‐mm stainless‐steel bar was inserted into a hole prepared in the piston body as the lug, and a rubber stopper, 3 mm in diameter, was attached at the end of the piston body. The piston was then placed into the barrel with the lug inserted in the notch. The outlet and inlet valves were assembled in the barrel. The button and piston springs were placed on the tops of the barrel and piston, respectively. On top of the springs, the button was placed with the lug inserted in the lug guide. The button cover was made by bonding a ring, cover film of polyurethane, and cover body, into which the button was inserted to make it seamlessly covered, finally producing the actuator. The drug container was made of a flexible polyurethane membrane, one end of which was assembled with a connector to be connected with the barrel in the actuator, and the other was assembled with a refill port containing a rubber septum. This assembly was then inserted into the reservoir cover to produce the drug reservoir. Complete assembly of the actuator and drug reservoir produced the BCD, and its entire outer surface was then coated with Parylene C for biocompatibility after implantation.^25^

### 2.3 In vitro performance test

To test the performance of the BCD in vitro, the BCDs (*n* = 3) were each filled with 0.92 ml of an exenatide solution (5 mg ml^−1^), and clicked while being fully immersed in 30 ml of pH 7.4 PBS at 37°C. The exenatide concentration in the medium was measured using high‐performance liquid chromatography (HPLC; 1260 series, Agilent Technologies), using a C18 column (5 μm, 4.6 mm × 250 mm; Agilent Technologies) at 25°C. The mobile phase of acetonitrile and water (containing 0.1% TFA) was delivered at a flow rate of 1 ml min^−1^ with a linear gradient elution, in which the acetonitrile content was 20% for the first 2 min and gradually increased to 80% until 6 min. The injection volume and UV absorbance were set at 10 μl and 215 nm, respectively.^26^, ^27^

### 2.4 Animals

For in vivo evaluation, we used 7‐week old female Wistar rats (Orient Bio) and male Zucker Diabetic Fatty (ZDF, Charles River) rats. The animals were housed in a pathogen‐free facility with controlled temperature, humidity, and 12:12 h light/dark cycle. The Wistar rats were provided with standard chow and water ad libitum. The ZDF rats were fed with Purina #5008 (Ralston Purina) chow and water ad libitum. To implant the BCD, an animal was anesthetized via isoflurane inhalation, and the dorsal area was shaved and sterilized with betadine. A 3‐mm incision was made, and the BCD was implanted into the subcutaneous pocket. The wound was closed with a surgical suture and disinfected with betadine. All animal experimental procedures were approved by the Institutional Animal Care and Use Committee at Seoul National University Hospital Biomedical Research Institute (approval no.: 19‐0070‐C1A1).

### 2.5 Pharmacokinetic studies on exenatide delivery

For the pharmacokinetic (PK) tests, the Wistar rats were divided into two groups: (1) animals treated with subcutaneous injections of exenatide (Inj‐Ex), and (2) animals implanted with the exenatide‐loaded BCD (BCD‐Ex), to which 50 μg of exenatide was administered via subcutaneous injection or button click, respectively. At 0, 15, 30, 60, 120, and 180 min after administration, 200 μl of blood was drawn from the tail vein. The blood sample was centrifuged at 3000*g* for 10 min to collect the plasma, which was then stored at −20°C until measurement. The exenatide concentration in blood plasma was measured using an exenatide enzyme immunoassay (EIA) Kit (Merck Millipore).

### 2.6 Gastric emptying rate measurement

We used the ZDF rats to compare the gastric emptying rate after 28 days of treatment in four different groups: (1) animals treated with subcutaneous injections of PBS without exenatide (Inj‐PBS, n = 3); (2) animals implanted and clicked with PBS‐loaded BCD without exenatide (BCD‐PBS, n = 3); (3) animals treated with subcutaneous injections of exenatide (Inj‐Ex, n = 4), and (4) animals implanted with the exenatide‐loaded BCD (BCD‐Ex, n = 4). For all animals, 10 μl of 5 mg ml−1 exenatide solution or PBS was administered twice per day for 28 days. Each animal was fasted overnight and then a 50 mg kg^−1^ paracetamol solution was administered orally. Blood was collected 0, 30, 60, 120, and 180 min after administration, and the blood plasma was obtained by centrifugation at 3000*g* for 10 min. Then, 10 μl of collected plasma was mixed with 90 μl of methanol, and 50 μl of the supernatant was transferred and evaporated in a SpeedVac (Thermo Fisher Scientific) under vacuum. The residue was dissolved in a mixture of DI water and acetonitrile (75:25, vol/vol), which was measured by HPLC mass spectrometry (LCMS) (6120 series, Agilent Technologies) with a C18 column (5 μm, 4.6 mm × 250 mm, Agilent Technologies). A mobile phase composed of DI water adjusted to pH 2.5 and acetonitrile (75:25, vol/vol) was fed at a continuous flow rate of 0.5 ml min^−1^, and the detection wavelength was set at 207 nm. Mass spectrometric detection (Agilent 6120 Quadrupole LCMS system) was performed with an Ion Spray source set at 350°C and 4000 V. Electrospray ionization in positive ion mode was employed, and quantification was performed by selected‐reaction monitoring transition (152 ➝ 153).

### 2.7 Oral glucose tolerance tests

For the oral glucose tolerance tests, we used the ZDF rats after 28 days of treatment in four different groups: Inj‐Ex, Inj‐PBS, BCD‐Ex, and BCD‐PBS. The animal was fasted overnight and a glucose solution (1 g kg^−1^) was administered by oral gavage. At scheduled times after administration, blood was collected, and blood plasma was obtained by centrifugation at 3000*g* for 10 min. The plasma glucose level was measured with a glucometer (Accu‐chek Performa). The plasma insulin level was measured with an insulin ELISA Kit (Merck Millipore).

### 2.8 PD studies on insulin and glucagon delivery

For this experiment, we employed a Type 1 diabetic animal model, streptozotocin (STZ)‐induced diabetic rats.^26^, ^28^ To induce this animal model, Wistar rats were fasted overnight, and then intraperitoneally injected with a STZ solution (60 mg kg^−1^) prepared in 0.09 M citrate buffered saline (pH 4.0) to destroy the beta‐cells of the pancreas. Three days after the STZ injection, the plasma glucose level was measured with a glucometer, and the animals with a plasma glucose level > 400 mg dl^−1^ were screened for use. Among them, we employed two distinct animal models of hyperglycemia and hypoglycemia, respectively. For a hyperglycemic condition, the animals were used as they were with a blood glucose level of >400 mg dl^−1^.^28^, ^29^ For a hypoglycemic condition, the animals were fasted overnight and the ones with a plasma glucose level of <72 mg dl^−1^ were screened for use.^30^ The hyperglycemic animals were treated with 1‐U insulin by an SC injection or implanted BCD: Inj‐Insulin (*n* = 4) and BCD‐Insulin (*n* = 4), respectively. For this, we utilized a commercially available insulin solution (100 U ml^−1^; NovoRapid, Novo Nordisk). The hypoglycemic animals were treated with 100‐μg glucagon by SC injections or an implanted BCD: Inj‐Glucagon (*n* = 4) and BCD‐Glucagon (*n* = 4), respectively. To prepare a glucagon solution (1 mg ml^−1^), glucagon powder (Glucagon HypoKit, Novo Nordisk) was dissolved in sterile DI water. After administration, the plasma glucose level was measured with a glucometer at scheduled times.

### 2.9 Histology

To examine the implant safety, the animals in the BCD‐Ex group were sacrificed by carbon dioxide inhalation, and the tissue was harvested from four different locations around the BCD: adjacent to the button, outlet, refill port, and wall (Figure 7). The tissue was fixed in 4% paraformaldehyde and embedded in paraffin wax, which was then cut into 4‐μm‐thick slices. Each tissue slice was then mounted on a glass slide, which was stained with hematoxylin and eosin (H&E). The slides were assessed by a professional pathologist (C. L.) in a blinded manner, using an optical microscope at ×200 and ×100 magnification (BX54; Olympus). For each tissue location surrounding the BCD, at least three images were assessed from each animal of the BCD‐Ex.

### 2.10 Analysis of epididymal fat pads

To examine the efficacy of the exenatide, the epididymal fat pads were biopsied at the end of the experiments. The tissue was fixed in 10% formalin and embedded in paraffin, which was sectioned into 4‐μm‐thick slices. Slices of adipose tissue were mounted on glass slides and stained with H&E.^31^ The slides were observed with an optical microscope at ×150 and ×100 magnification. For one animal from each group, three slides were randomly selected, and the average size of the adipocytes was measured using ImageJ software (National Institute of Health).

### 2.11 Statistical analysis

The data were presented as means ± SD. Statistical analysis was performed using GraphPad Prism 7 (GraphPad Software). The differences among the groups were tested using one‐way analysis of variance (ANOVA) or two‐way ANOVA followed by Tukey's honestly significant difference post hoc test for multiple comparisons. To compare the means of two groups, Student's *t* tests were used. Differences were considered to be statistically significant when *p* < 0.05.

## 3 RESULTS

### 3.1 BCD design

**FIGURE 1:**
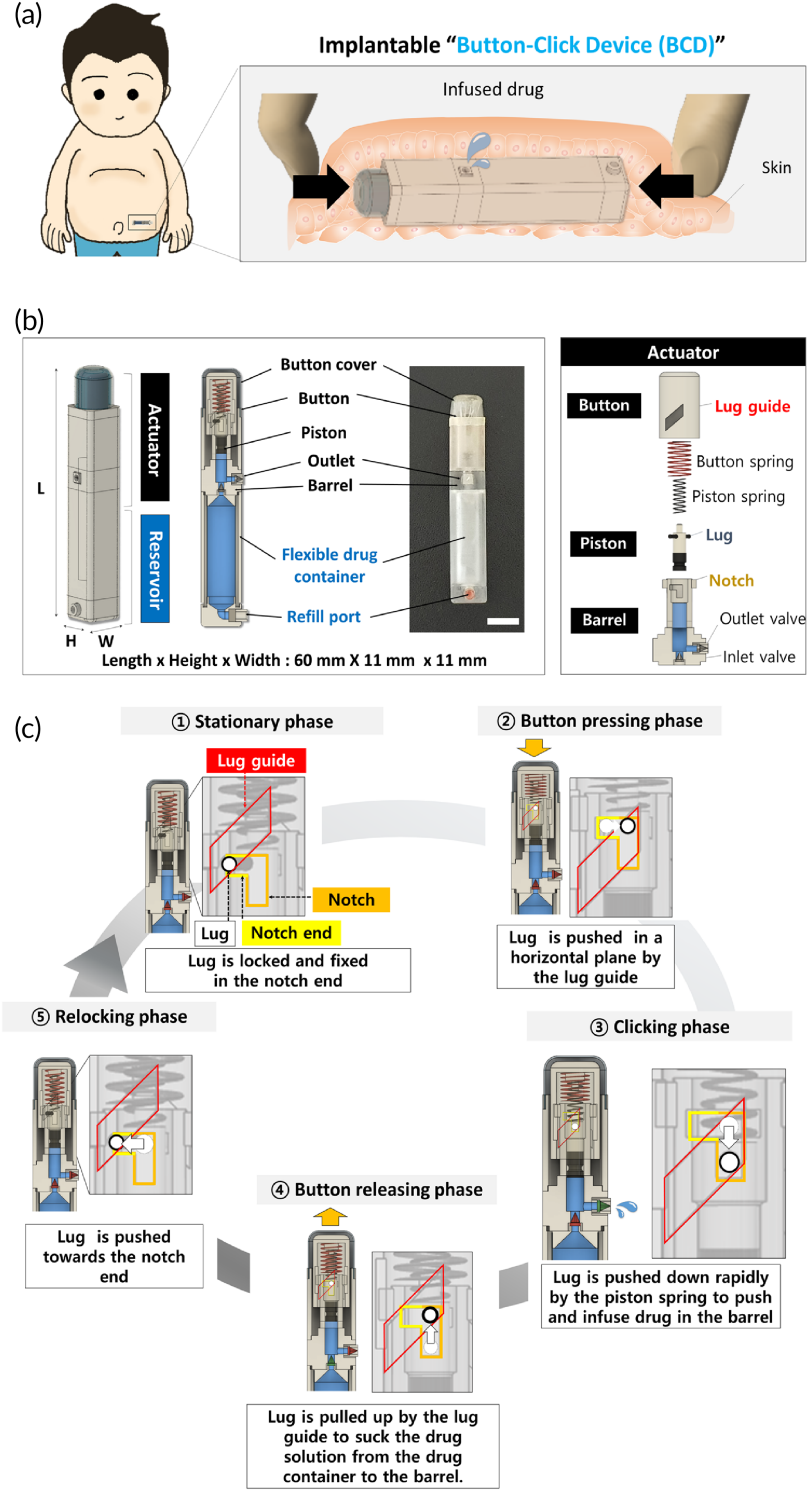
Description of the BCD. (a) Schematic illustration depicting drug infusion by button clicks on the skin above the BCD implanted subcutaneously. (b) 3D schematic and optical images of the BCD. Scale bar = 1 cm. (c) 3D schematic illustration, showing the working principle of the BCD (more detailed illustrations are available in Figure S2 and Movie S1)

The BCD was designed to deliver a specific amount of drug only when the button in the implanted BCD is pressed by fingers through the skin. Therefore, as depicted in Figure 1a, we designed the BCD as a rod shape with a button at one end. The dimensions are: length 60 mm, height 11 mm, and width 11 mm. The device is short enough for an adult to grip the BCD and press the button with a thumb and an index finger.^32^ The incision for implantation is minimized by the use of a rod shape with a relatively small cross‐section. We envision that the BCD could be implanted subcutaneously, under the skin of the abdomen or forearm, to be easily accessible for manual button‐clicking.

As shown in Figure 1b (also Figure S1), the BCD consists of two major parts: (1) an actuator with a button cover, button, piston, and barrel, and (2) a reservoir that has a drug container and refill port that are adhered and connected seamlessly. In the actuator, the button is seamlessly covered with a button cover, to prevent infiltration of biological fluids or tissues into the button. The film in the button cover is made of flexible polyurethane, to allow the button to be pushed and clicked. The button and piston are connected with two springs in parallel: the button and piston springs. The diameter of the piston spring fits that of the piston, and the button spring has a larger diameter, to account for the inner diameter of the button. There is a lug in the piston, which is inserted in the lug guide formed in a see‐through diagonal line in the button wall. The barrel mainly works as an intermediate container, from which a drug can be sucked from the drug container and infused out through the inlet and outlet valves. There is a vertical notch in the barrel, into which the lug in the piston is inserted. In the drug reservoir, the drug container stores the drug needed for multiple infusions. The drug reservoir is made of a flexible polyurethane membrane, which is designed not to produce a negative pressure during drug consumption.^26^ The refill port protrudes slightly, and is easily detected and localized on the thin skin layer above the implanted BCD.^33^ Through this refill port, fresh drug solution can be injected into the drug container, using a tiny needle (31G), while the BCD is still implanted.

### 3.2 BCD working principles

The working principle of the BCD described here is based on click actuation, as shown in Figure 1c (also see Figure S2 and Movie S1), which is obtained by two distinct springs and the guided movement of the piston, with the lugs inserted in both the lug guide in the button and the notch in the barrel. At the (1) stationary phase, the lug is locked in the notch end, so the piston is fixed in position. When the button starts to be pressed at the (2) button pressing phase, the button moves downward to compress both button and piston springs together. During this process, the lug guide formed in the button wall pushes the lug through the notch in a horizontal plane, thereby producing no downward, but only rotational movement of the piston. However, at a certain pressing depth of the button, the lug reaches the vertically bent point of the notch and is freed to start the (3) clicking phase. At this stage of click actuation, the button spring is still compressed, but the piston spring is decompressed quickly to push the piston downward. This action builds up a positive pressure in the barrel, which opens the outlet valve to infuse a specific volume of the drug solution, providing an accurate drug dose per click. At the (4) button releasing phase, the button spring is decompressed to move the button upward, and the lug guide pushes the lug through the notch to move the piston upward, creating negative pressure in the barrel. This action opens the inlet valve to suck the drug solution from the drug container and refill the barrel. At the (5) relocking phase, the button is continuously released, and the lug reaches the vertically bent point of the notch and starts to rotate in a horizontal plane. The lug finally gets into the notch end to lock and fix the position of the piston, returning back to the (1) stationary phase.

The click actuation of the BCD is a response to a sudden, rapid downward movement of the piston when the piston spring is decompressed, with the lug freed from the notch end. This response occurs only above the predetermined button pressing depth, and at this depth, the piston spring is compressed to the same extent. Therefore, for every click actuation, the piston spring is decompressed to deliver the same pushing force to the barrel, thereby producing an accurate, reproducible amount of drug infusion (Movie S2). This response is also a safety feature, because the BCD does not click and infuse the drug when the button is pressed part of the way. A click produces a sudden decrease in force at the button (Movie S3), which provides a tactile sensation that indicates drug infusion. We used a spring that operates at a compression pressures between the cutaneous pressure threshold (1.09 ± 0.25 N cm^−2^) and the pain threshold (32.1 ± 15.3 N cm^−2^).^34^ Therefore, the button cannot be pressed accidentally, and the pain can be minimized when the button is pressed through the skin (Figure S3 and Movie S4).

### 3.3 In vitro performance tests

**FIGURE 2:**
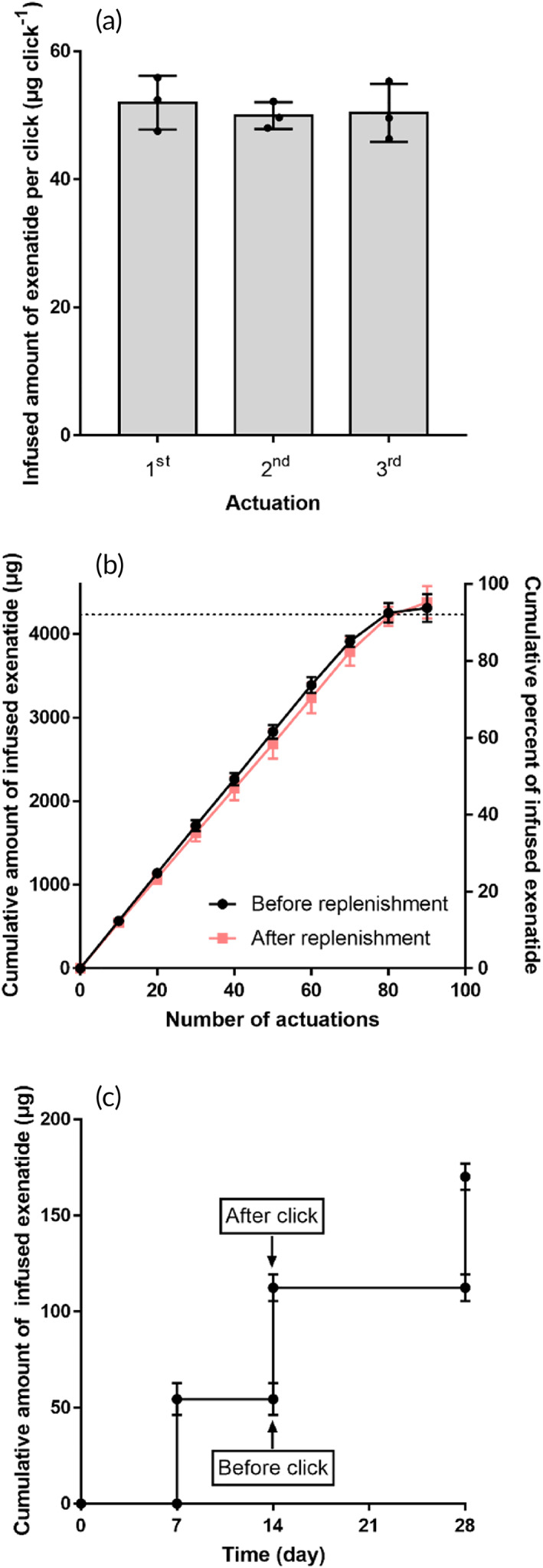
In vitro performance of the exenatide‐loaded BCD. (a) Infused amount of exenatide per click. The BCDs (*n* = 3) were each clicked three times while being fully immersed in pH 7.4 PBS at 37°C. (b) Accelerated depletion test results before and after replenishment. The BCDs (*n* = 3) were clicked repeatedly until completely depleted. The BCDs were then replenished with fresh solution, after which, the accelerated depletion test was repeated. An accurate amount of exenatide was infused per click until almost all of the drug was consumed (ca. 94%), which was reproducible after replenishment. Data are means ± standard deviation (SD). (c) Long‐term profile of exenatide infusion. The exenatide‐loaded BCDs (*n* = 3) were each clicked at 7, 14, and 28 days while being fully immersed in pH 7.4 PBS at 37°C for 4 weeks. There was no release of exenatide when the button was not pressed

To test the performance of the BCD, we filled the drug container with a solution of a self‐injection drug, exenatide,^35^, ^36^ and clicked the BCD under in vitro environments, as described in Figure 2a. With each click, a predetermined amount of exenatide was released (50.8 ± 3.2 μg per click), demonstrating the capacity of the BCD to infuse an accurate volume of liquid by click actuation (10.2 ± 0.2 μl drug solution per click) (Movie S2). When the BCD was clicked more than once (Figure 2b), a reproducible profile of drug infusion could be maintained until around 94% of the exenatide was consumed, because the drug reservoir was made of a flexible material, preventing a negative pressure build‐up.^26^ This reproducibility was not affected after drug replenishment. To evaluate the feasibility of long‐term use of the system, the BCD was fully immersed in PBS for 4 weeks, and clicked at scheduled times. As shown in Figure 2c, the BCD released a reproducible amount of infused drug only when clicked, and there was no drug leakage observed between clicks. This accurate dose was still reproducible when the BCD was actuated up to 1000 times consecutively (Figure S4). During this 4‐week period, the stability of exenatide and insulin was observed to be well retained (Figure S5).

### 3.4 In vivo experiments for exenatide delivery

We first verified the in vivo performance of the BCD for the delivery of exenatide, and compared it with the conventional approach to delivery, subcutaneous injection. We used four groups of Type 2 diabetic/obese animals, ZDF rats Inj‐PBS (*n* = 3), BCD‐PBS (*n* = 3), Inj‐Ex (*n* = 4), and BCD‐Ex (*n* = 4). For the Inj‐Ex and BCD‐Ex, the same dose of exenatide (50 μg) was administered twice per day for 28 days. Clicking the button of the BCD implanted under the skin did not appear to cause significant pain or discomfort to the animals during the testing period (Figure S3 and Movie S4).

**FIGURE 3:**
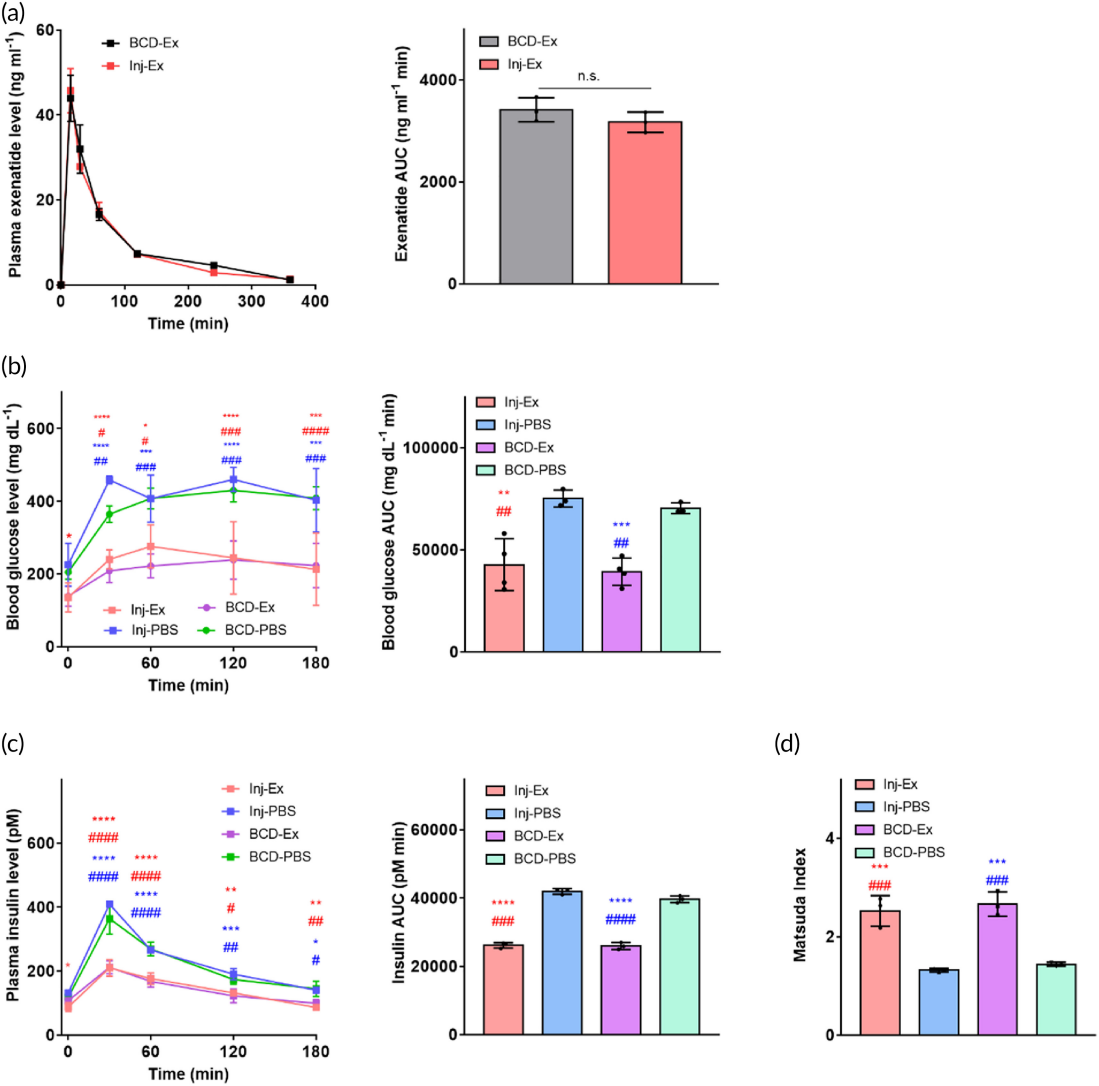
In vivo efficacy of the BCD for exenatide delivery. (a) Pharmacokinetic profiles of exenatide. Oral glucose tolerance tests were performed on ZDF rats after 28 days of treatment, showing the profiles of (b) blood glucose level, (c) plasma insulin level, and (d) Matsuda index. The AUC was calculated using the trapezoidal rule. Data are means ± SD. Red *; Inj‐Ex versus Inj‐PBS, Blue *; BCD‐Ex versus Inj‐PBS, Red #; Inj‐Ex versus BCD‐PBS, and Blue #; BCD‐Ex versus BCD‐PBS. *p < 0.05, **p < 0.01, ***p < 0.001 and ****p < 0.0001. #*p* < 0.05, ##*p* < 0.01, ###*p* < 0.001, and ####*p* < 0.0001

Figure 3a shows the PK profile of exenatide in the Inj‐Ex and BCD‐Ex. This difference was not statistically significantly different, producing similar maximum plasma exenatide concentrations (*C*_max_) and time to reach *C*_max_ (*T*_max_) for both groups. Thus, the areas under the plasma drug concentration‐time curve (AUC) were also similar, at 3170 ± 162.9 ng ml^−1^ min for the Inj‐Ex and 3414 ± 193.2 ng ml^−1^ min for the BCD‐Ex. When the BCD was clicked after replenishment with a fresh exenatide solution while still implanted (Figure S6 and Movie S5), the PK profile did not change (Figure S7), suggesting that the replenishment procedure did not affect the performance of the BCD.

After 28 days of treatment, the PD profile of exenatide was examined. We first assessed glucose metabolism by conducting an oral glucose tolerance test in all four animal groups.^37^ As shown in Figure 3b, the groups treated with exenatide, Inj‐Ex, and BCD‐Ex, had a significantly smaller increase in plasma glucose level than those treated with PBS, Inj‐PBS, and BCD‐PBS.^38^ Thus, the AUC of plasma glucose was statistically significantly lower in the exenatide groups. When the plasma insulin was assessed (Figure 3c), however, the exenatide groups had lower insulin secretion than the PBS groups, producing a higher Matsuda index (Figure 3d). Those results indicate that long‐term treatment with exenatide enhanced insulin sensitivity in Type 2 diabetic and obese animals similarly in both Inj‐Ex and BCD‐Ex. This could be ascribed to the effect of exenatide, which is known to reduce hepatic insulin resistance by increasing beta‐cell mass.^39^

**FIGURE 4:**
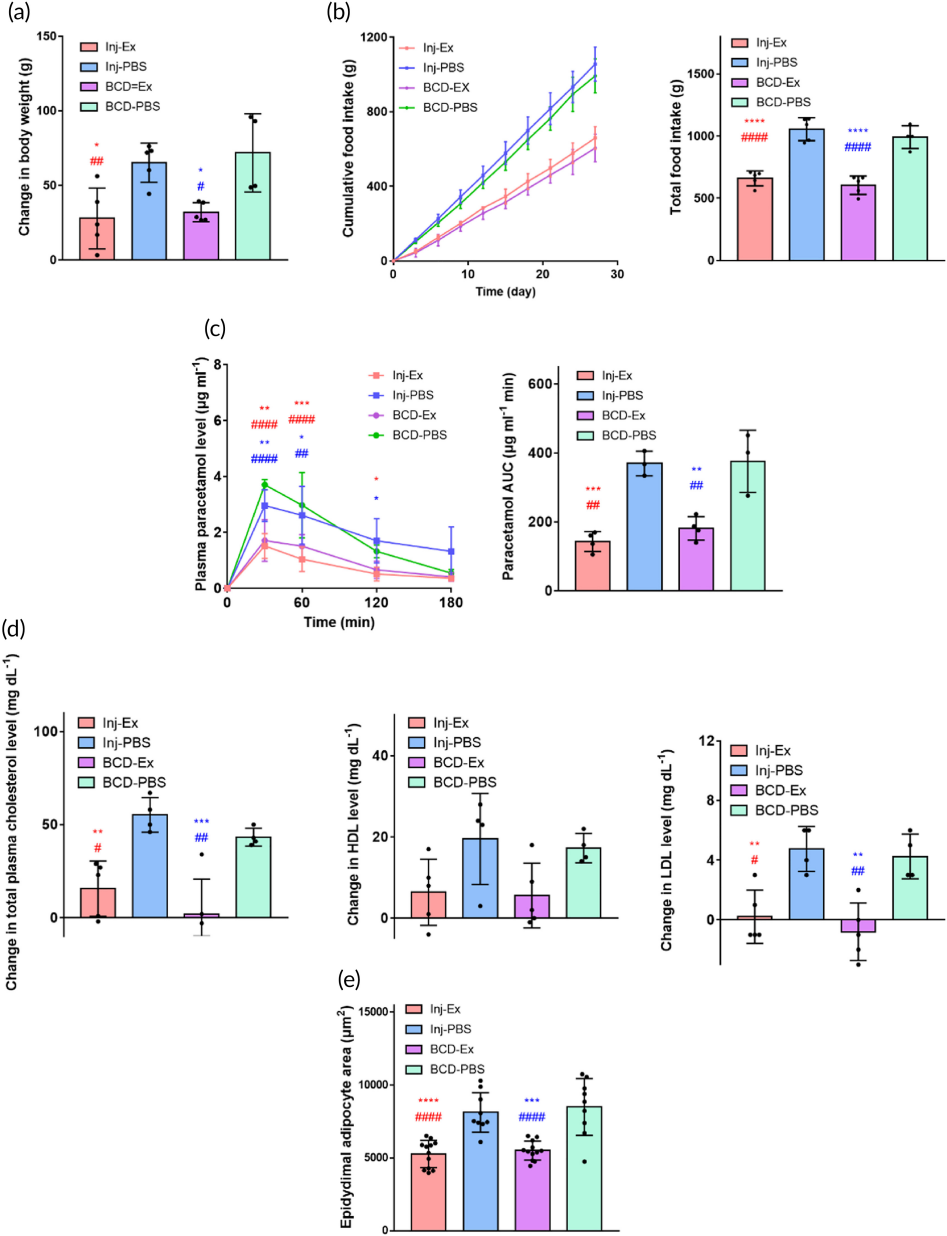
In vivo efficacy of the BCD for exenatide delivery in obesity tested on ZDF rats. Profiles of (a) body weight change and (b) food intake amount. (c) Gastric emptying rates assessed by the profiles of plasma paracetamol levels after oral administration. The AUC was calculated using the trapezoidal rule. (d) Changes in total cholesterol, HDL, and LDL levels before and after 28‐day experiment. For each animal, a blood sample was collected before and after 28 days of treatment, and was measured with a clinical chemistry analyzer (Hitachi‐7180, Hitachi). (e) Results of epididymal adipose tissues biopsied at the end of experiments, where the sizes of adipocytes were compared among the groups. Representative images of H&E‐stained epididymal adipose tissue are available in Figure S6. Data are means ± SD. Red *; Inj‐Ex versus Inj‐PBS, Blue *; BCD‐Ex versus Inj‐PBS, Red #; Inj‐Ex versus BCD‐PBS, and Blue #; BCD‐Ex versus BCD‐PBS. *p < 0.05, **p < 0.01, ***p < 0.001 and ****p < 0.0001. #*p* < 0.05, ##*p* < 0.01, ###*p* < 0.001, and ####*p* < 0.0001

With respect to other PD parameters of exenatide, both Inj‐Ex and BCD‐Ex exhibited a significantly lower increase in body weight and food intake than the PBS groups (Figure 4a,b).^40^, ^41^ The exenatide groups also had a slower gastric emptying rate than the PBS groups, and therefore had lower levels of insulin secretion and lower postprandial plasma glucose levels (Figure 4c).^42^ The PD profiles were similar in the Inj‐Ex and BCD‐Ex groups, indicating that the BCD could deliver exenatide with similar efficacy as conventional subcutaneous injections, and that the animals coped well with BCD implantation and multiple button clicks on the skin.

In this obese animal model, we also evaluated the changes in plasma cholesterol level before and after 28 days of treatment. As shown in Figure 4d, the exenatide groups had a decrease in total plasma cholesterol levels, with a higher decrease in the plasma level of low‐density lipoprotein (LDL).^43^ The plasma level of high‐density lipoprotein (HDL), albeit different on average, was not statistically significantly different between the exenatide and PBS groups. We assessed the epidydimal fat pads biopsied from the animals (Figures 4e and S8),^44^ and found that the average size of the adipocytes was smaller in the exenatide groups. Again, the effects of exenatide on the obese animals were similar between the Inj‐Ex and BCD‐Ex groups.

### 3.5 In vivo experiments on insulin and glucagon delivery

The fully implantable BCD described here is designed to deliver drugs on‐demand, using noninvasive button clicks over the skin. In this aspect, the BCD can be also used for delivery of other self‐injection drugs, such as insulin or glucagon. In general, prandial insulin is injected two or three times daily before each meal,^45^ and glucagon injection is used to treat severe hypoglycemia.^46^ To examine the applicability of the BCD to these situations, we used a Type 1 diabetic animal model, STZ‐induced diabetic rats, with hyperglycemia or hypoglycemia (Figure S9). For hyperglycemia, insulin was delivered by subcutaneous injections or BCD button clicks, respectively: Inj‐Insulin (*n* = 4) and BCD‐Insulin (*n* = 4). For hyperglycemia, glucagon was delivered by subcutaneous injections or BCD button clicks, respectively: Inj‐Glucagon (*n* = 4) and BCD‐Glucagon (*n* = 4).

**FIGURE 5:**
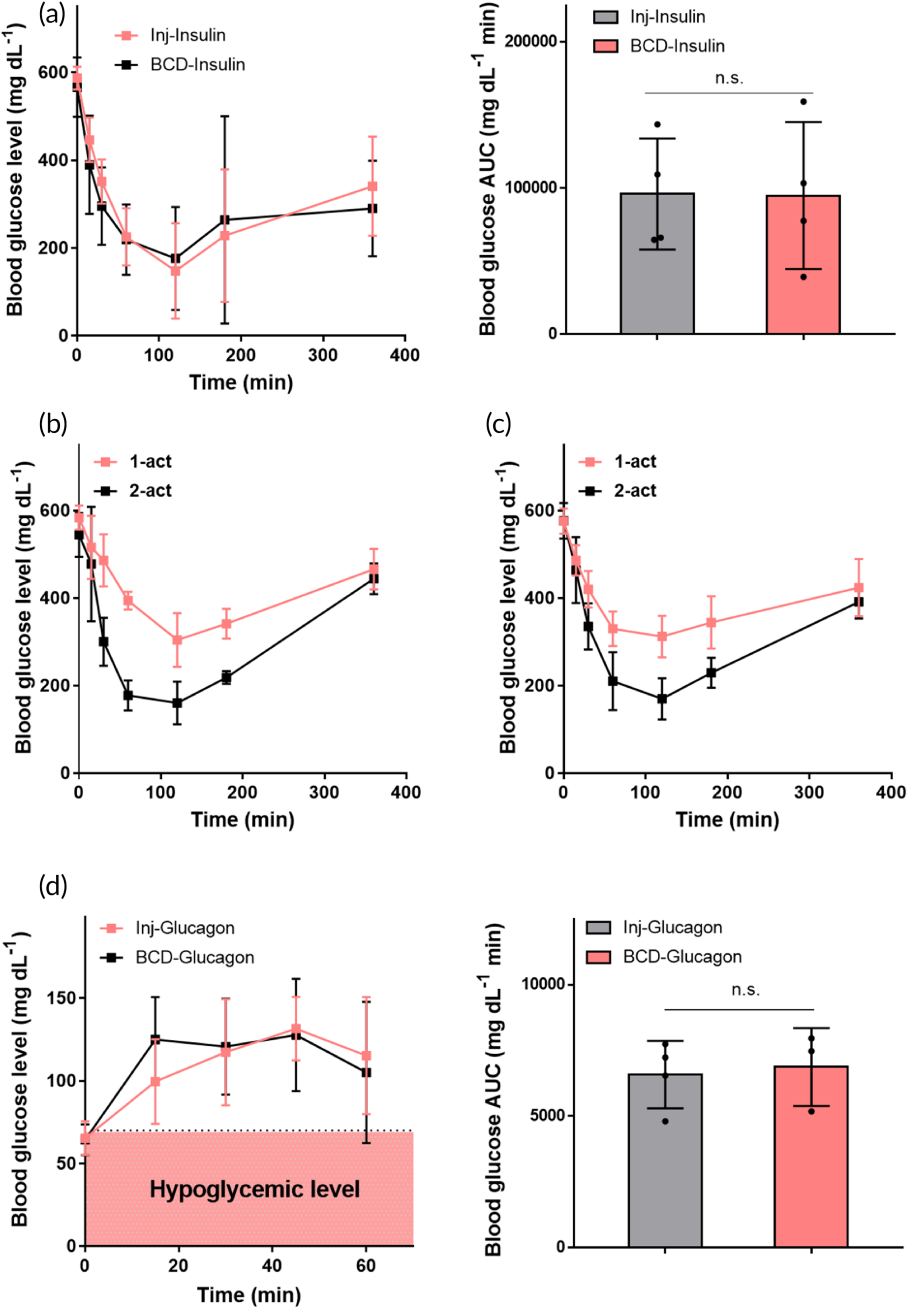
In vivo efficacy of the BCD for insulin or glucagon delivery. Profiles of blood glucose level after administration of (a) insulin and (b) glucagon were examined under hyperglycemic or hypoglycemic conditions of STZ‐induced Type 1 diabetic rats, respectively. For each condition, the profiles were not significantly different between the injection and BCD groups (*p* > 0.05). Data are means ± SD. The AUC was calculated using the trapezoidal rule

**FIGURE 6:**
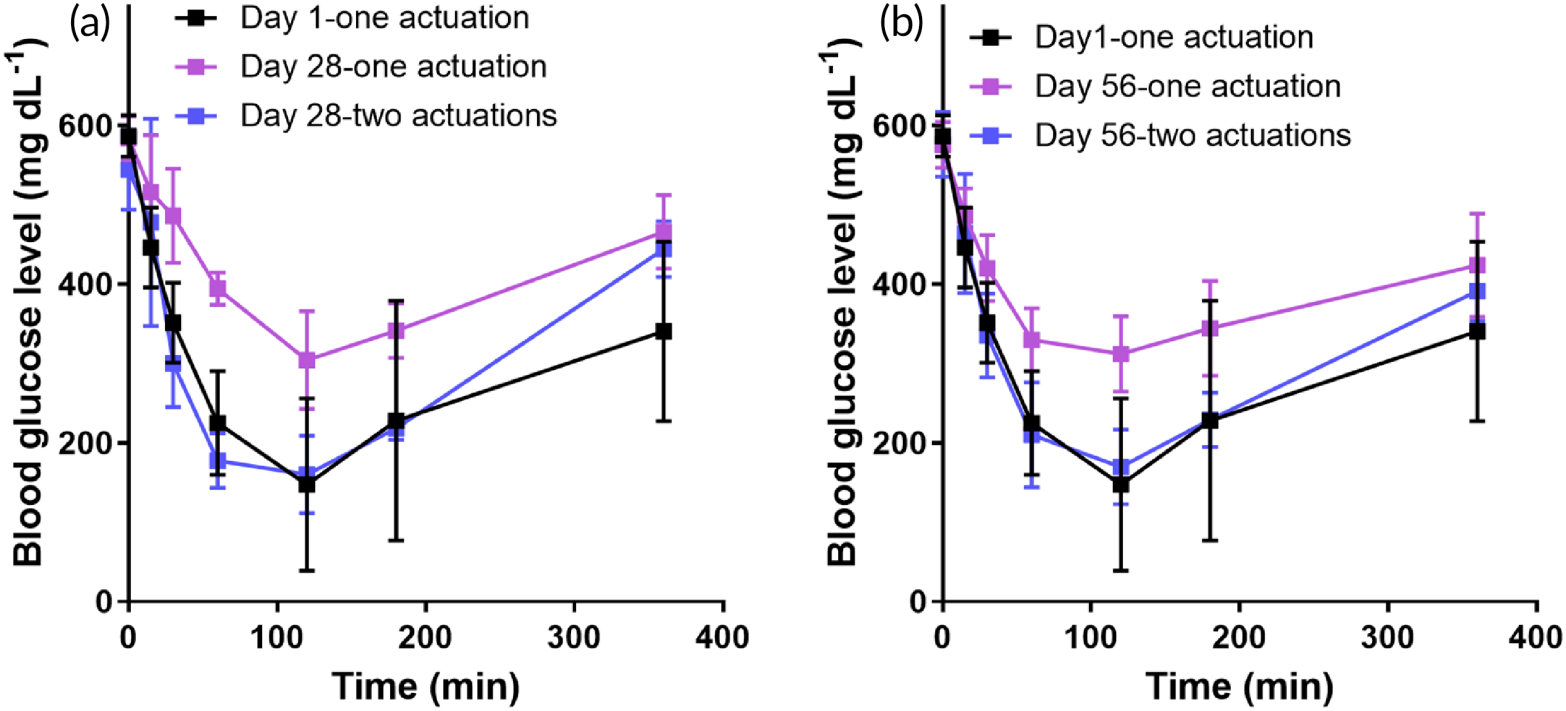
In vivo long‐term efficacy of the BCD pump. For this, the BCD loaded with insulin was implanted for 56 days (*n* = 3), where the profile of blood glucose level after administration of insulin was examined under hyperglycemic conditions of STZ‐induced Type 1 diabetic rats on (a) Day 28 and (b) Day 56 after BCD implantation. When the BCD was actuated once, the blood glucose level did not decrease as much as that treated in the same way on Day 1. This could be due to the formation of fibrotic capsule around the implanted BCD,^22^ which appeared to hinder systemic exposure of insulin released locally from the BCD. However, this could be compensated by actuating the BCD twice in a row, where the blood glucose dropped similarly to the one treated with a single actuation on Day 1. The profile of blood glucose level was not different between Days 28 and 56, implying a stabilized fibrotic capsule thickness without further growth

In the Type 1 diabetic model, we assessed the changes in plasma glucose levels. When hyperglycemic, animals administered insulin showed a decrease in plasma glucose level (Figure 5a). An increase in plasma glucose level was measured when glucagon was delivered to hypoglycemic animals (Figure 5b). For both insulin and glucagon delivery, the profile and AUC of the plasma glucose level were similar between the groups treated with subcutaneous injections and those treated using BCD button clicks, indicating that the BCD with noninvasive button clicks can be widely applied to replace self‐injection. To give an insight of a long‐term use, the BCD loaded with insulin was implanted for 56 days, where the efficacy of insulin delivery was examined (Figure 6). From Day 28, when the BCD was actuated once, systemic exposure of insulin did not appear to be as efficient as the one observed on Day 1 possibly due to a fibrotic tissue formation around the implanted BCD.^22^ However, this could be compensated simply by increasing the number of actuations, that is, two consecutive actuations, and double the local dose of insulin. The profiles of blood glucose level did not vary from Day 28, implying a stabilized capsule thickness without further growth.

### 3.6 Biocompatibility assessment

**FIGURE 7:**
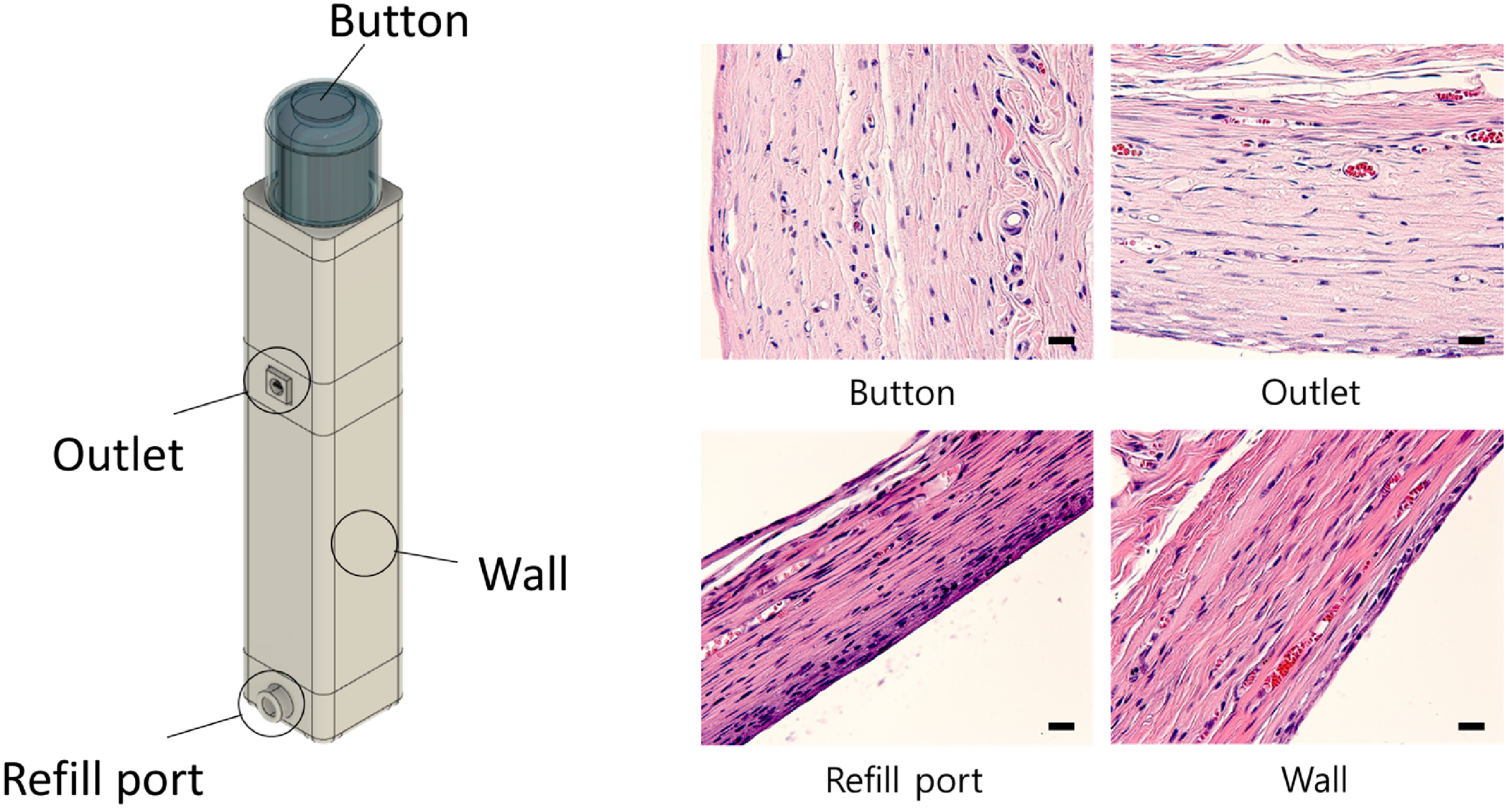
Representative histological images of the tissues surrounding the BCD. The tissues adjacent to each location in the BCD were separately obtained and assessed on H&E staining. Images were obtained with an optical microscope at ×200 magnification. Scale bars = 20 μm

To assess the biocompatibility of the device, we performed a histological analysis of the tissues surrounding the implanted BCD, with the tissues biopsied at the end of in vivo experiments (the BCD‐Ex). The tissues adjacent to the button, outlet, refill port, and wall of the BCD were prepared separately, stained with H&E, and assessed by a professional pathologist (C. L.) (Figure 7). In all tissues, there was an insignificant degree of inflammation, and few polymorphonuclear cells were observed (Figure 7). This observation suggested that the implanted BCD was biocompatible, and that multiple button presses did not affect nearby tissues. There was no tissue growth or sign of blockage in the outlet, indicating the feasibility of continuous drug administrations with the implanted BCD. When implanted for a longer period of 56 days, a fibrotic capsule formation was apparent (Figure S10), which would maintain the location and orientation of the BCD after implantation. The thickness was measured to be about 220 μm, which was thinner than that reported with other clinically available bulky implants.^47^ The fibrotic capsule did not increase further from Day 28 as observed in previous studies.^22^

## 4 DISCUSSION

Recent developments in self‐injectable therapies have transformed our ability to effectively treat widespread metabolic disorders.^1^, ^2^ In particular, deficiency or abnormally low secretion of large proteins can be compensated for by bolus administration, maintaining homeostasis.^48^, ^49^ With self‐injection, this can be achieved through home‐care, without frequent hospital visits,^50^ and therefore could be beneficial for chronic disease needing long‐term medication.^6^ However, multiple needle injections are painful and inconvenient for patients, and therefore, poor medication adherence is a problem.^51^ A jet injector can be considered an up‐to‐date technique for less painful self‐injections; however, there could be some discomfort due to a possible skin puncture with high‐pressurized liquid injections.^52^, ^53^

An implantable drug delivery device has been suggested as a promising alternative to frequent injections.^22^, ^26^, ^54^ To mimic a circadian secretion profile and allow for an on‐demand, pulsatile administration of bolus drugs, many devices operate with sophisticated control units, such as electronic actuators and circuit components driven by batteries, which makes them relatively large for implantation, and requires repeated surgeries due to a limited battery lifetime.^19^, ^20^, ^55^ Some devices were designed to operate without batteries, having noninvasive stimuli applied from outside the body, such as magnetic fields,^22^, ^26^ light,^21^, ^56^, ^57^ and ultrasound,^58^, ^59^ to actuate the implanted device to release drugs. However, even in those cases, patients may always need to wear or carry an additional device that can provide the stimulus needed to administer drugs. This can be considered similar to the situation, carrying prefilled pens, syringes or injectors for conventional self‐injection^52^, ^60^ (Figure S11). To address these problems, we developed a fully implantable BCD, capable of an on‐demand bolus drug administration with easy manual operation, using button clicks on the skin. The BCD does not require an additional controller to be carried for drug administration. Instead, the button of an implanted BCD can be found and clicked on the skin to infuse the drug solution only when needed. The drug dose can easily be modulated by varying the number of button clicks (Figure 2). As the BCD infuses a specific volume of liquid, the drug dose per click can also be varied by changing the concentration of the drug solution in the reservoir. The button clicks are not painful, but are perceptible with a proper selection of the button and piston springs (Figure S3 and Movie S4). The BCD is equipped with a refill port, which is shaped to be protruded, and thus, it can be easily located from the outside skin. Through the refill port, the drug solution can be replenished using a tiny needle (31G) while the BCD is still implanted (Figure S6 and Movie S5). The refill port is made of a rubber septum, as already used in other implantable devices in clinical use,^61^, ^62^ which can be tightly re‐sealed when the needle is retracted. This replenishment procedure does not affect the reproducibility of drug infusion (Figure S7). Hence, there is no need for BCD replacement surgery, allowing semi‐permanent use after implantation.

To allow an accurate amount of drug infusion, we used click actuation, rather than simple squeezing, for infusion. During button press and release, the lug in the piston is guided in a designated path and controlled distance set in the notch, so the pressure change in the barrel can be accurate and reproducible (Figures 1 and S2, and Movie S1). This feature could be further controlled using the outlet and inlet valves in the barrel. Thus, at the moment of button click, an accurate pressure increase is obtained in the barrel, where the drug solution is infused, just to compensate for the pressure threshold set in the outlet valve. When the button is released, the piston moves backward the same distance, to create a specific decrease of pressure in the barrel, which can be refilled with a drug solution to compensate for the pressure threshold set in the inlet valve. Because of those valves, the infiltration of biological fluid can be prevented, and the fluid in the barrel cannot flow into the main drug reservoir.

Click actuation also adds safety features to the BCD. Once clicked, the button must be released completely to fill the barrel and pressed again to provide a dose of the drug. Thus, even when the button is fully pressed and maintained, an unwanted overdose, or continuous leak of drug is prevented. The notch is vertically‐bent to form the notch end, where the lug can be locked to fix the piston in position when the button is not pressed (Figure 1c). Because of this intentional restriction, even if the button is pressed halfway by mistake, the piston rotates only in a horizontal plane, and the pressure in the barrel does not increase, so no drug leaks through the outlet valve.

We found that the BCD could deliver an accurate amount of a drug, such as exenatide, insulin, or glucagon, which was as effective as conventional injections in an animal model of metabolic disease (Figures 3, 4, 5). Animals implanted with the exenatide‐loaded BCD exhibited in vivo PK and PD profiles similar to those treated with injections (Figures 3 and 4).^41^, ^63^ Prandial insulin and glucagon could also be accurately delivered with the BCD, again attaining similar PD profiles to those produced by injection (Figure 5). All constituent units of the BCD were packed into a seamless device, and the entire outer surface was coated with biocompatible Parylene C.^64^ Thus, the tissues around the implanted BCD exhibited no abnormal foreign body responses (Figure 7).

For clinical application, several improvements to the prototype of the BCD should be made. To be accepted by the patients, the body of the BCD may need to be round and cylindrical in shape, to minimize the sensation of a foreign body. The BCD needs to be compact while still containing enough drug solution that replenishment is only infrequently needed. A clinically approved implantable insulin pump that recommends replenishment every 3 months contains about 13–15 ml of the drug in the reservoir.^65^ Thus, we envision a slightly larger, cylindrical BCD, with a cross‐sectional area up to 3 cm^2^ (i.e., diameter = 2 cm) and a length of about 8 cm. This would increase the length of the drug container up to 5 cm, while the actuator could remain around 3 cm, as in the prototype in this study, because the BCD can be operated with simple button clicks without complex control units and batteries, hence a small fixed volume of the actuator. Therefore, this entire dimension could still be much smaller than the clinically approved implantable pump (diameter = ca. 8 cm and thickness = ca. 2 cm).^65^ Under this condition, at most 40 times of a replenishment procedure is expected considering 10 years of BCD use.

For better patient convenience, the use of a mobile app can be envisioned to notify the time for replenishment,^66^, ^67^, ^68^ which can be programmed to alarm periodically at the times when the drug in the reservoir was not yet fully consumed, based on the prescribed regimen. This schedule can be arranged together with regular hospital visits needed for the patients with chronic diseases,^69^, ^70^, ^71^, ^72^ where the clinicians can replenish the drug in the implanted BCD (Figure S6). For safety, the drug reservoir needs to be firmly protected from any possible rupture. In this sense, the outer case of BCD should be made from a biocompatible metal of high mechanical strength, such as titanium or stainless‐steel.^73^, ^74^ If these considerations were met, the BCD could be used to replace currently available administration strategies for many different self‐injectable medications (Figure S11, such as interferon, gonadotropin‐releasing *hormone*, peripheral vasodilators, anabolic hormones, etc.^75^, ^76^, ^77^, ^78^

## 5 CONCLUSIONS

In summary, we have developed a semi‐permanent, fully implantable BCD capable of on‐demand bolus drug administration with easy manual button clicks, to replace needled self‐injection therapies. After a single implantation, the BCD can infuse a precise amount of a drug only when clicked with the fingers, noninvasively, from outside the body. Our in vivo experimental results indicated that use of the BCD produced pharmacokinetic profiles and the PD effects equivalent to those produced by subcutaneous injection. Therefore, we concluded that the semi‐permanent BCD is a promising treatment platform, transforming the clinical landscape of self‐injectable therapies in a noninvasive way.

## CONFLICT OF INTERESTS

Yong Chan Cho, Cho Rim Kim, Seung Ho Lee, and Young Bin Choy are listed as inventors on the pending patents (PCT/KR2020/015827 and KR 10‐2020‐0009763) filed by SNU R&DB for the button‐click devices described in this article. This technology has been transferred to ToBios Inc., where Y.B.C. is a founding member. The other authors declare no conflict of interests.

## AUTHOR CONTRIBUTIONS

**Cho Rim Kim:** conceptualization, methodology, validation, investigation, data curation, and writing—original draft. **Yong Chan Cho:** conceptualization, methodology, validation, investigation, data curation, and writing—original draft. **Seung Ho Lee:** conceptualization, methodology, validation, investigation, data curation, and writing—original draft. **Jae Hoon Han:** validation and investigation. **Min Ji Kim:** validation and investigation. **Han Bi Ji:** validation and investigation. **Se‐Na Kim:** validation and investigation. **Chang Hee Min:** validation and investigation. **Byung Ho Shin:** validation, investigation, data curation. **Cheol Lee:** methodology, validation, investigation, data curation. **Young Min Cho:** methodology, validation, investigation, data curation. **Young Bin Choy:** conceptualization, methodology, supervision, project administration, funding acquisition, and writing—original draft.

### PEER REVIEW

The peer review history for this article is available at https://publons.com/publon/10.1002/btm2.10320.

## Supporting information


**DATA S1** Supporting Information.

**Movie S1** Drug infusion by button clicks with the BCD.

**Movie S2** Reproducible liquid infusion by button clicks with the BCD.

**Movie S3** Profile of force applied on the button of the BCD during click actuation.

**Movie S4** Button click applied while the BCD was still implanted. When the BCD was clicked, the animal without anesthesia did not show any sign of pain or discomfort.

**Movie S5** Drug replenishment while the BCD was still implanted, using a 31 G syringe needle.

## Data Availability

The data that support the findings of this study are available from the corresponding author upon reasonable request.
