# Supplementary material for: Implantable device actuated by manual button clicks for noninvasive self‐drug administration
Source: Bioeng Transl Med. 2022 Apr 5;8(1):e10320. doi: 10.1002/btm2.10320 (PMC9842066; doi:10.1002/btm2.10320)
Supplement: Supplementary file 1 — DATA S1 Supporting Information. [file BTM2-8-e10320-s002.docx]

Supplementary Materials for

**Implantable Device Actuated by Manual Button Clicks for
Noninvasive Self Drug Administration**

Cho Rim Kim^†^, Yong Chan Cho ^†^, Seung Ho Lee^†^, Jae Hoon Han, Min Ji Kim, Han Bi Ji,

Se-Na Kim, Chang Hee Min, Byung Ho Shin, Cheol Lee, Young Min Cho,

and Young Bin Choy*

*Correspondence author. Email: [ybchoy@snu.ac.kr](mailto:ybchoy@snu.ac.kr)

^†^These authors contributed equally as first authors to this work.

**Table of contents**

**Supplementary Figure 1.** Detailed schematic description of BCD assembly procedure.

**Supplementary Figure 2.** Detailed schematic description of click actuations.

**Supplementary Figure 3.** Pressures measured for click actuation in the BCD, using various combinations of the button and piston springs.

**Supplementary Figure 4.** Mechanical stability assessment of the BCD by consecutive 1000 actuations.

**Supplementary Figure 5.** Stability assessment of exenatide and insulin in the BCD.

**Supplementary Figure 6.** Schematic description of replenishment procedure for the BCD pump.

**Supplementary Figure 7.** Pharmacokinetic profiles of the BCD before and after the replenishment of exenatide.

**Supplementary Figure 8.** Representative images of H&E stained epididymal adipose tissues biopsied at the end of experiments.

**Supplementary Figure 9.** Schematic description of experimental procedures for insulin and glucagon delivery.

**Supplementary Figure 10.** Histopathological image of the tissues around the BCD on (A) day 28 and (B) day 56 after implantation.

**Supplementary Figure 11.** Comparison table of administration strategies for self-injection drugs.

**Captions for Supplementary Movies**, 1 to 5


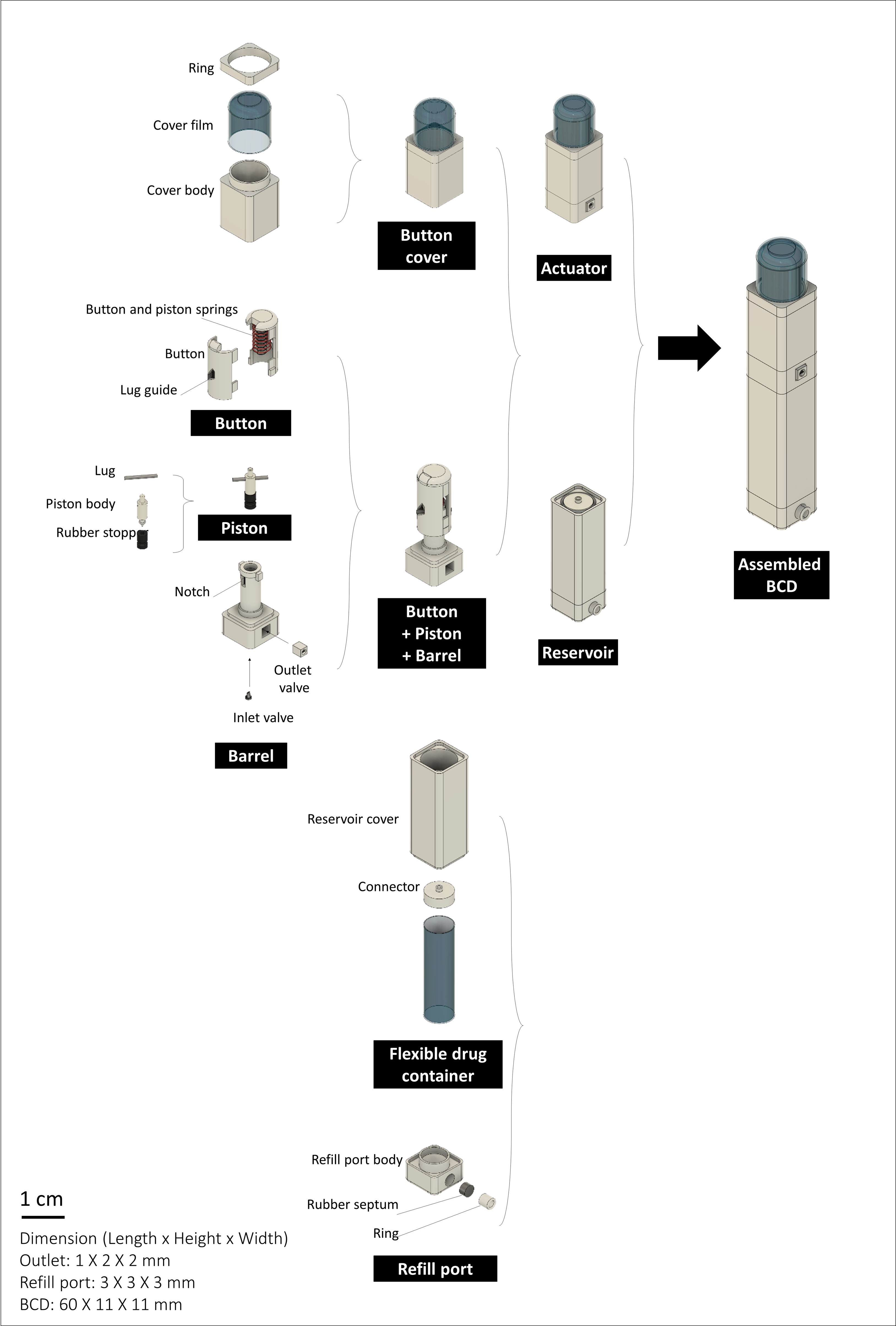


**Supplementary Figure 1. Detailed schematic description of BCD assembly procedure.**

**
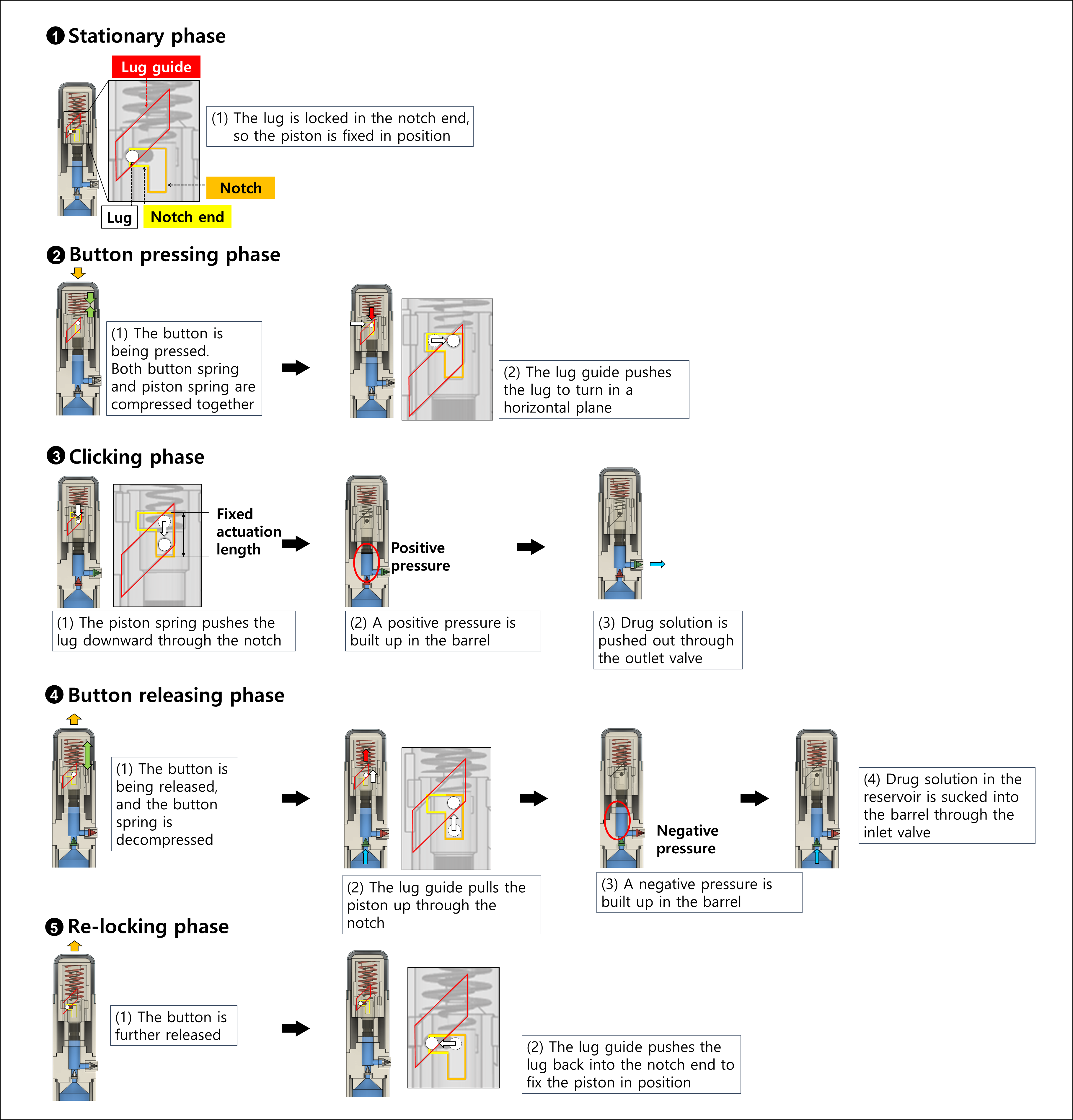
Supplementary Figure 2. Detailed schematic description of click actuations.**

**
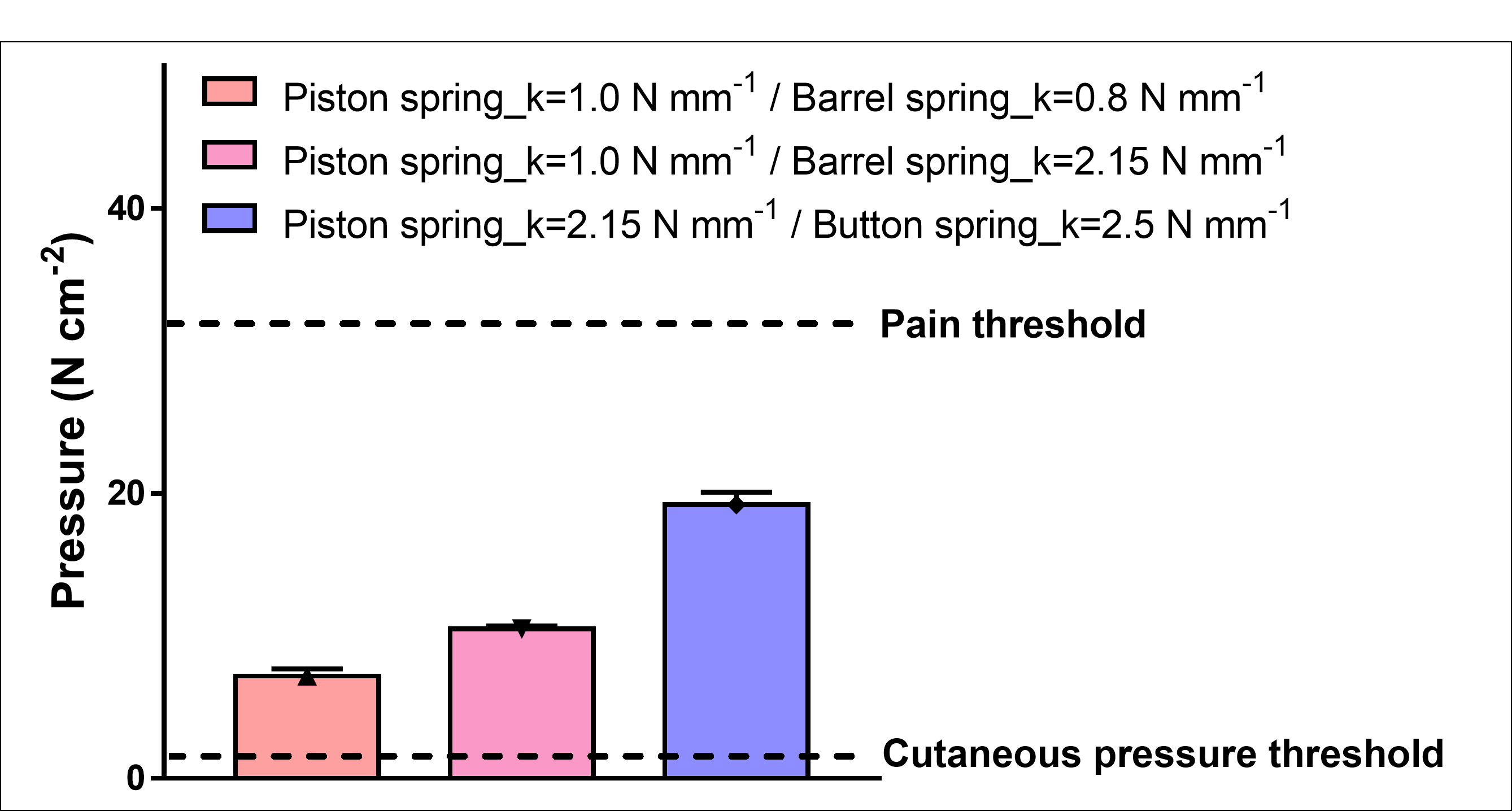
**

**Supplementary Figure 3. Pressures measured for click actuation in the BCD, using various combinations of the button and piston springs.** For each combination of springs with different spring constants, the button of the BCD was pressed with a micro-fatigue tester (E3000LT, Instron, United Kingdom), and the maximum pressure right before click actuation was recorded. Those results suggested that the click actuation for drug infusion could be obtained under a variety of pressures by employing various combinations of the springs, and the pressure range could be lower than the pressure pain threshold and higher than the cutaneous pressure threshold (the pressure perceived). We used a piston spring of k = 2.15 N mm^-1^ and a button spring of k = 2.5 N mm^-1^ in the BCD in this work (blue bar in the graph) (also see Supplementary Movie 3), where the pressure needed for BCD actuation is higher than a natural intraabdominal pressure generated in daily life (Cobb et al., 2005).

**Supplementary Figure 4. Mechanical stability assessment of the BCD by 1000 consecutive actuations.** The amount of exenatide infused per actuation was still accurate and reproducible, and there was no mechanical failure observed. Those 1000 actuations may be able to account for at least 500 days of BCD use in case where two administrations per day are needed.

**
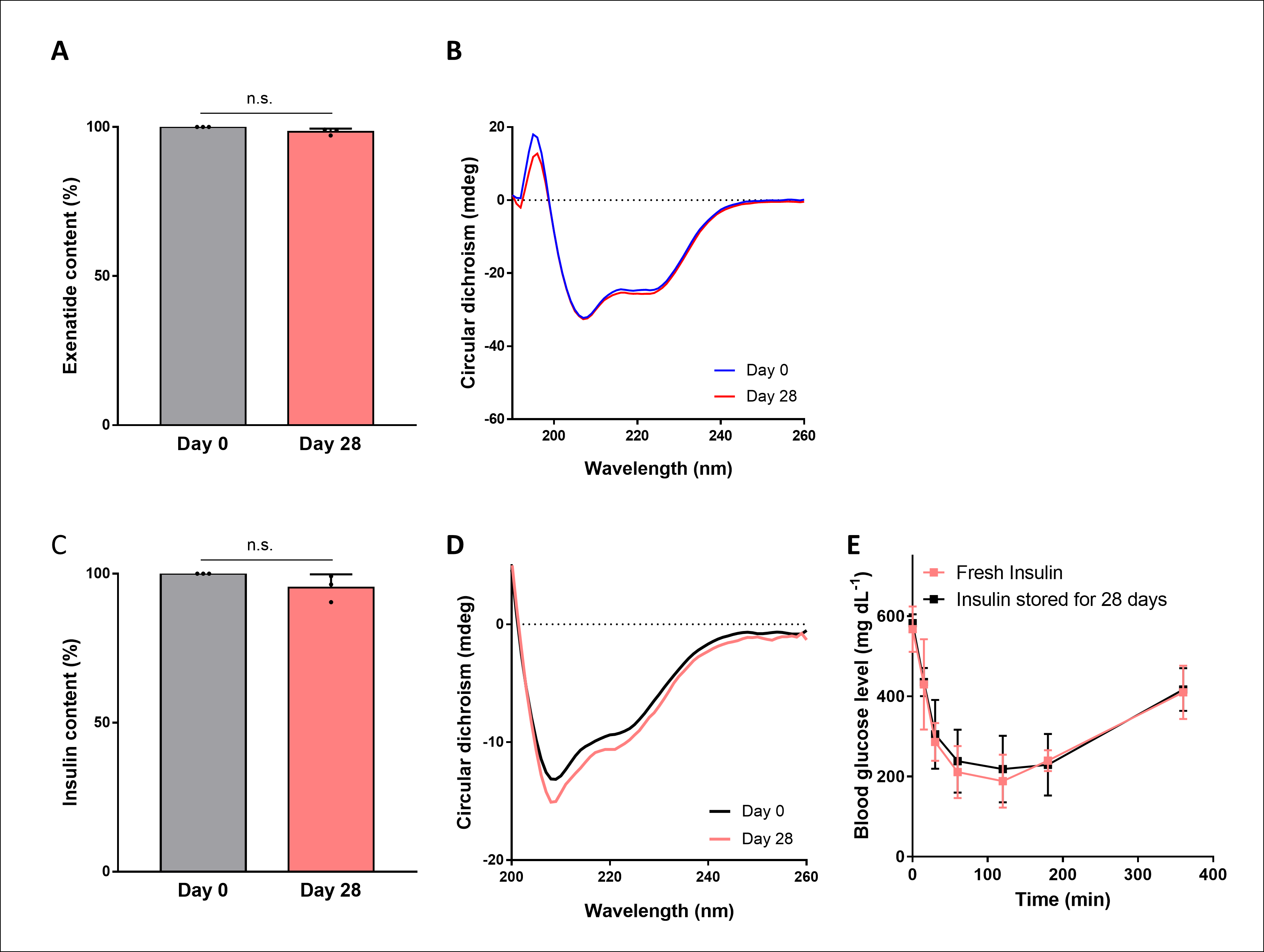
**

**Supplementary Figure 5. Stability assessment of exenatide and insulin in the BCD.** The exenatide or insulin-loaded BCDs (*n* = 3) were stored at 37 °C for 28 days, after which the solution in each BCD was extracted and compared with a fresh drug solution (n = 3). (A) HPLC and (B) Far-UV circular dichroism (CD) spectroscopy results from exenatide, and (C) HPLC and (D) Far-UV CD spectroscopy results from insulin. To obtain the CD spectra, the solution was placed in a 1-mm path length cuvette (Chirascan plus, Applied Photophysics, Surrey, UK) and scanned over range of 200-260 nm. The drug content and CD spectra did not vary after incubation, suggesting the retained stability of exenatide and insulin in the BCD at body temperature. (E) A fresh and stored insulin was injected subcutaneously to diabetic animals (n = 3), respectively, where the drop of blood glucose level was observed to be similar, suggesting the retained efficacy of insulin stored in the BCD at body temperature.


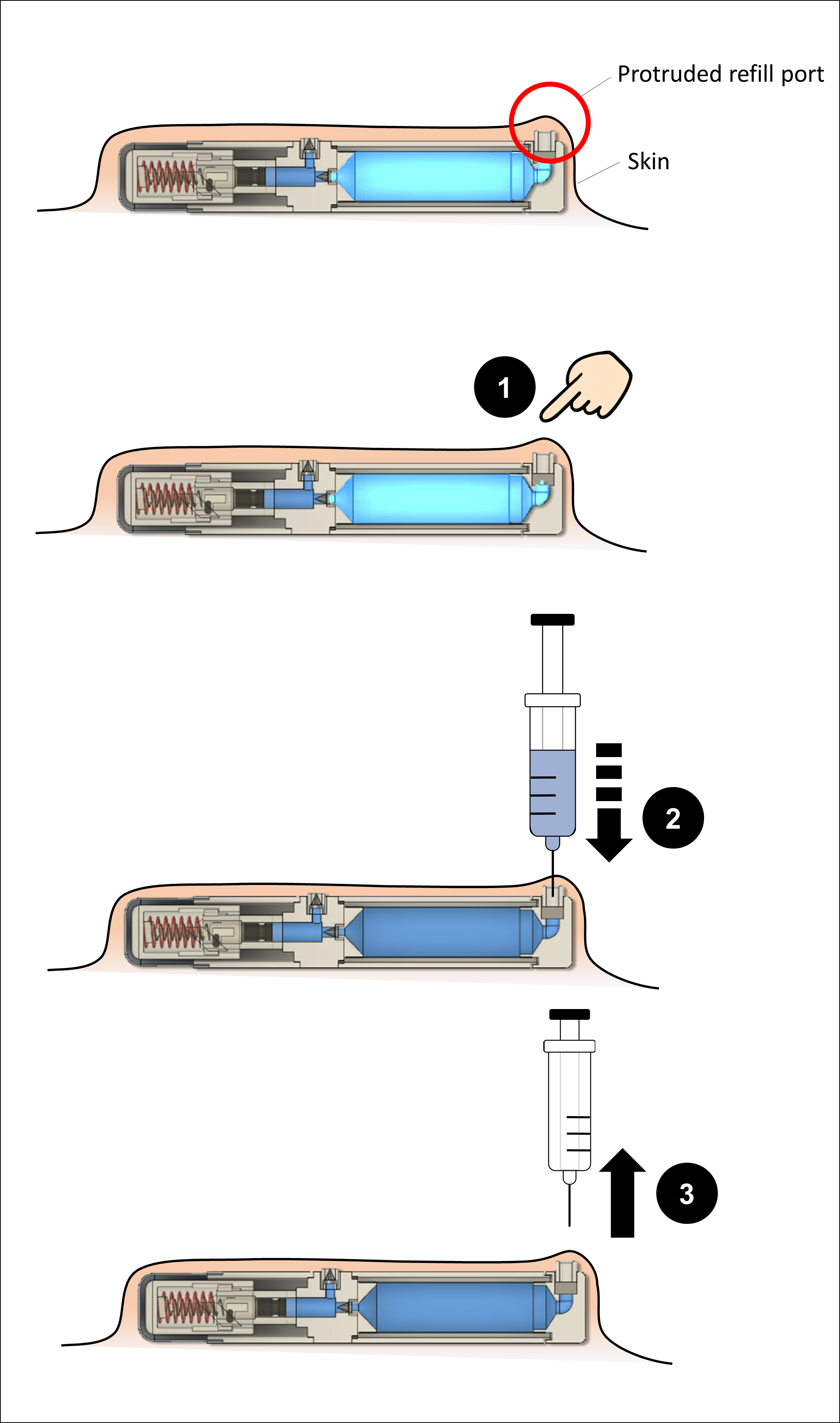


**Supplementary Figure 6. Schematic description of replenishment procedure for the BCD pump.** 1) Perceive and locate the protruded refill port in the BCD from the outside skin. 2) Insert the needle through a rubber septum in the refill port and slowly infuse a fresh drug solution into the BCD. 3) Retract the needle and a rubber septum is re-sealed in the refill port.


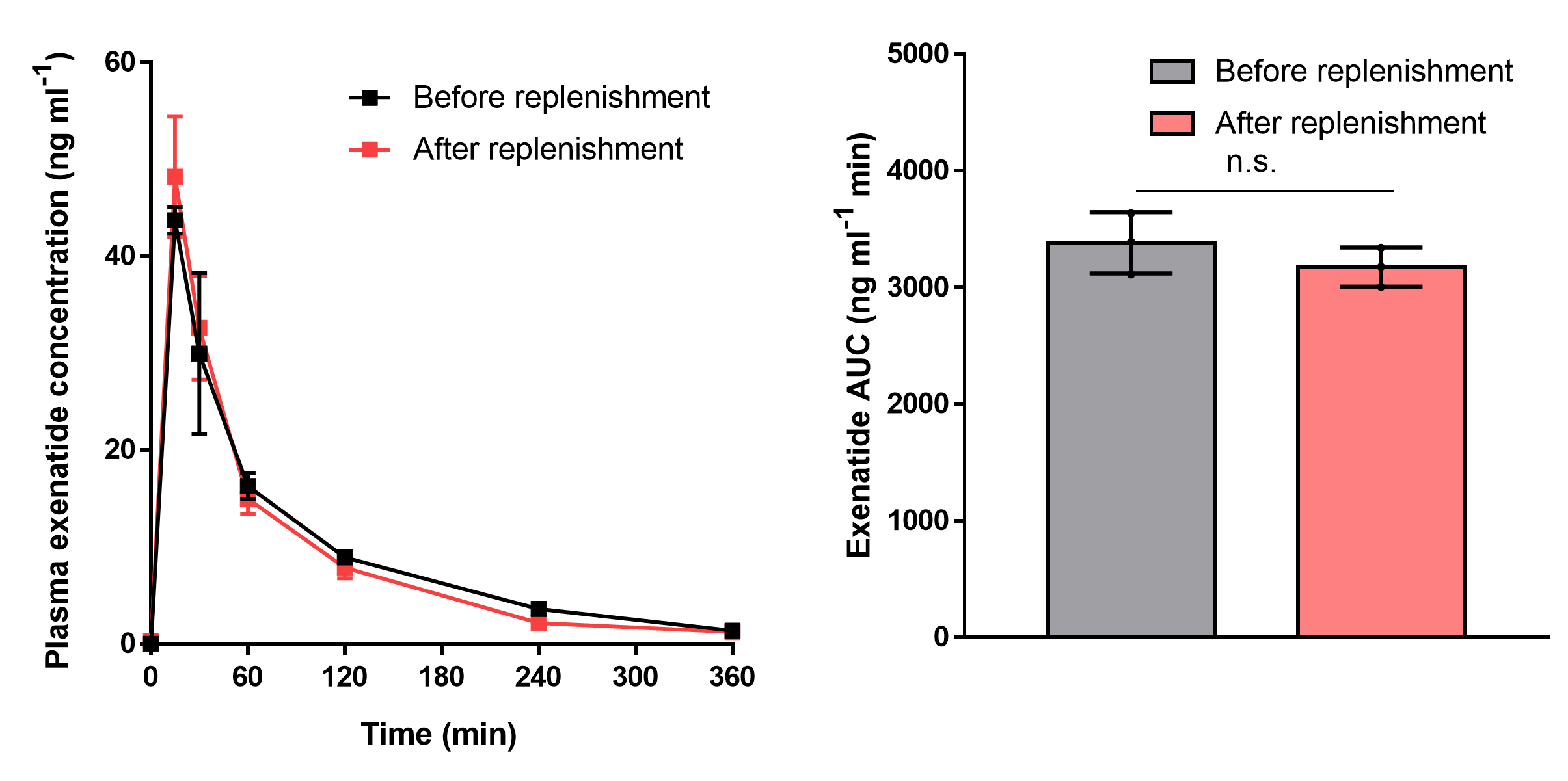


**Supplementary Figure 7. Pharmacokinetic profiles of the BCD before and after the replenishment of exenatide.** After the first PK test with the animals of BCD-Ex (Figure 3A), the BCD was replenished with a fresh exenatide solution (5 mg ml^-1^) while the BCD was still implanted (Supplementary Movie 5). We then clicked the BCD and performed the same PK studies. The PK profile and AUC of exenatide were unchanged even after replenishment.

**
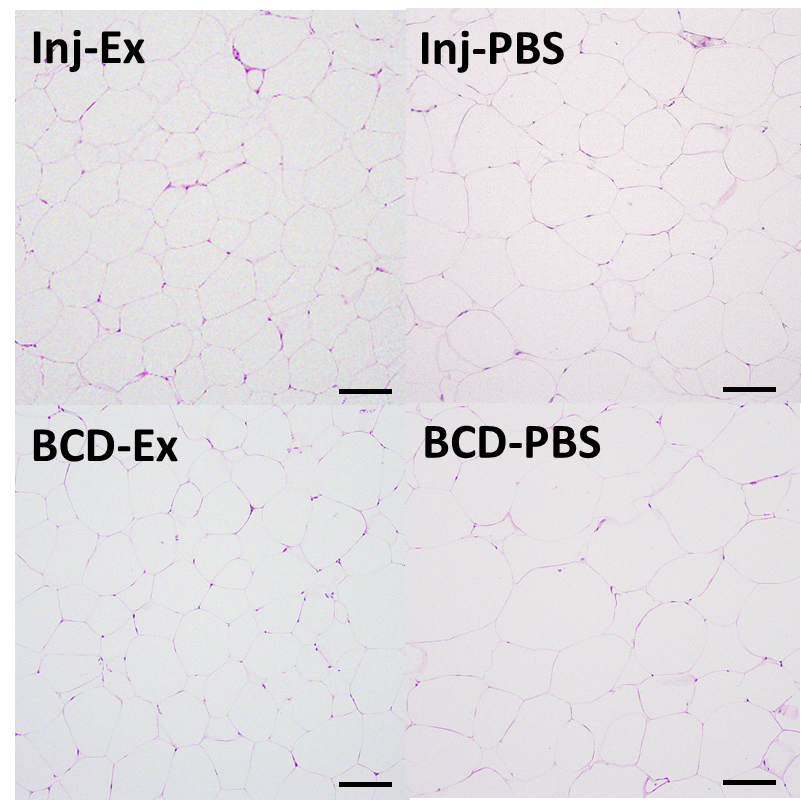
**

**Supplementary Figure 8. Representative images of H&E stained epididymal adipose tissues** **biopsied at the end of experiments.** The average size of the adipocytes was smaller in the exenatide groups than in the PBS groups. Scale bars = 100 *μ*m.

**
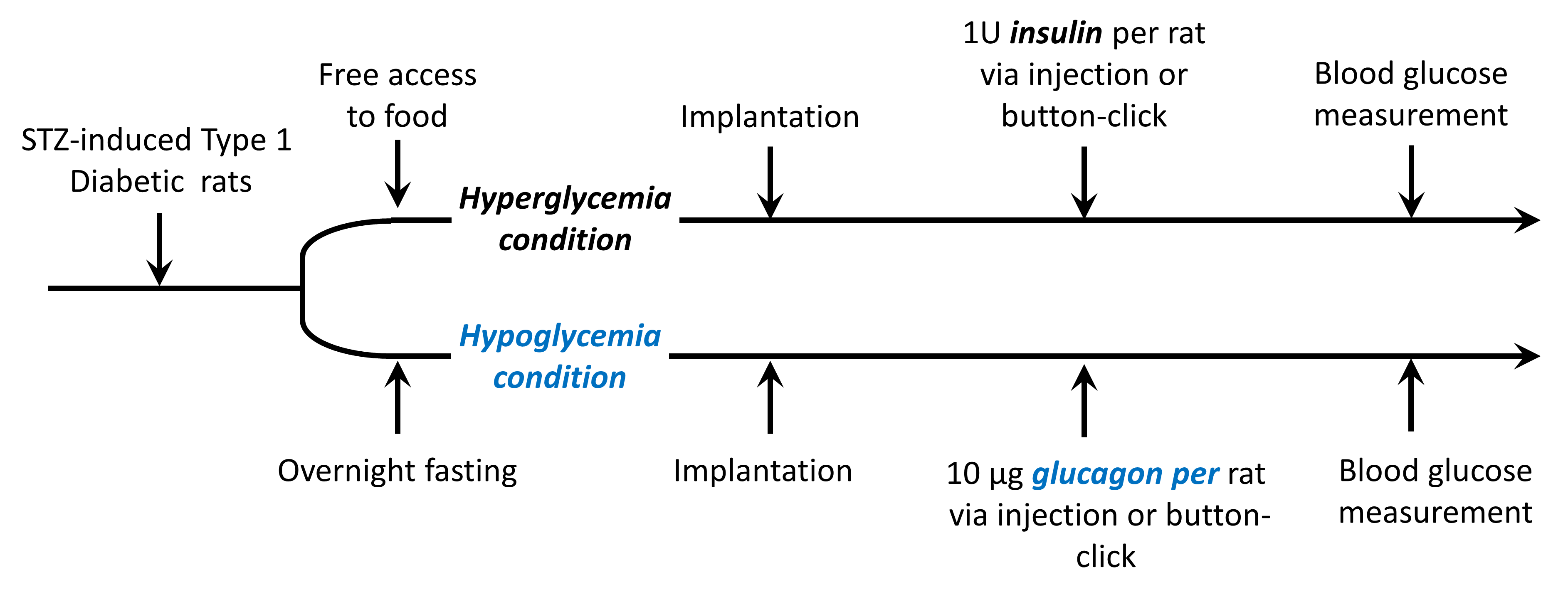
**

**Supplementary Figure 9. Schematic description of experimental procedures for insulin and glucagon delivery.**

**
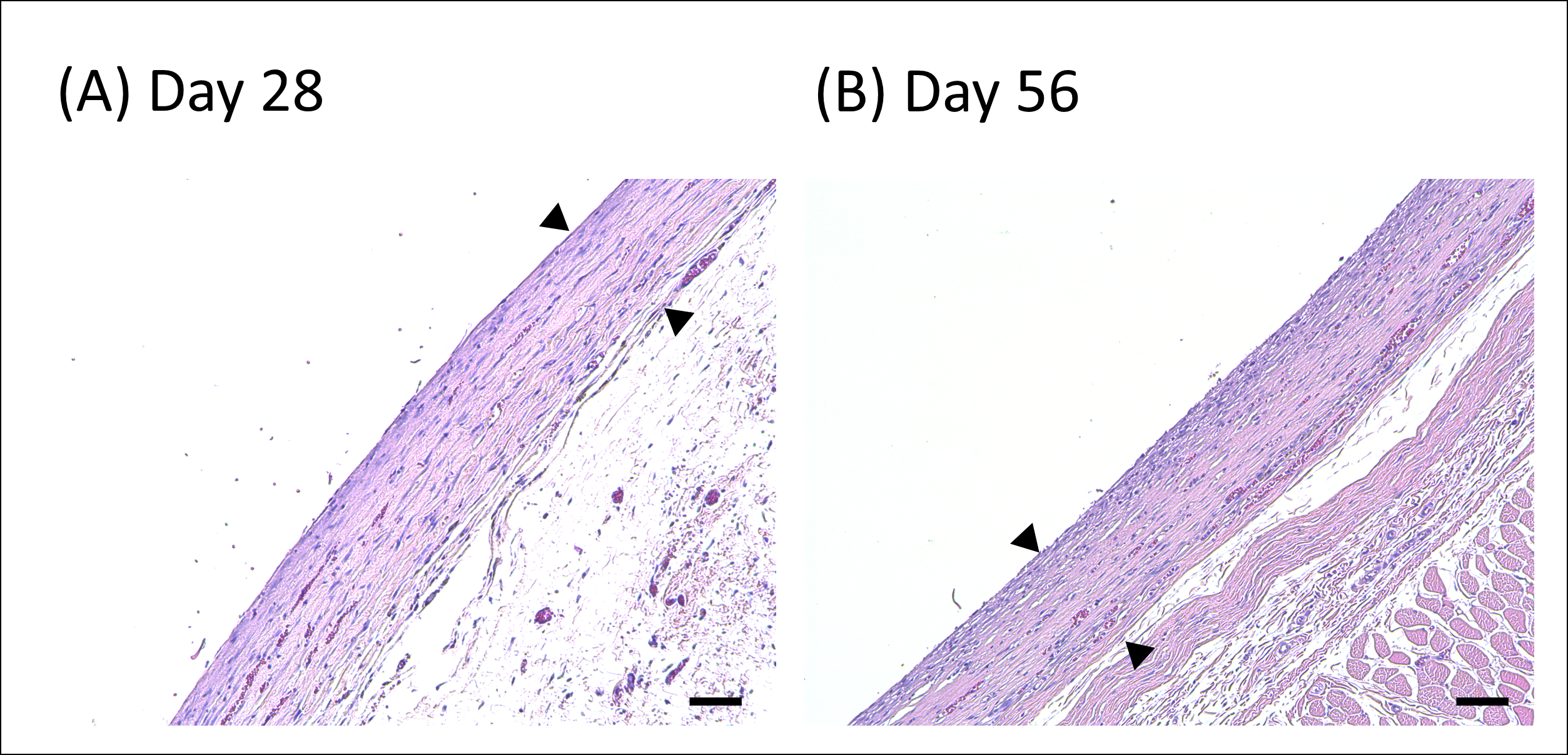
**

**Supplementary Figure 10. Histopathological image of the tissues around the BCD on (A) day 28 and (B) day 56 after implantation.** To assess the fibrotic capsule formed around the BCD, the tissues were biopsied and H&E-stained. On each day of biopsy, four tissue slides were randomly selected from each of three animals and thus, a total of twelve images were assessed. From each image, the thinnest region was selected and measured to give a capsule thickness (Jeon et al., 2018; Kim et al., 2017), as indicated by the arrows. There was almost no difference in capsule thickness between day 28 and 56, which were measured to be 215.20 ± 23.17 and 219.46 ± 26.77 μm, respectively. Scale bars = 100 μm.


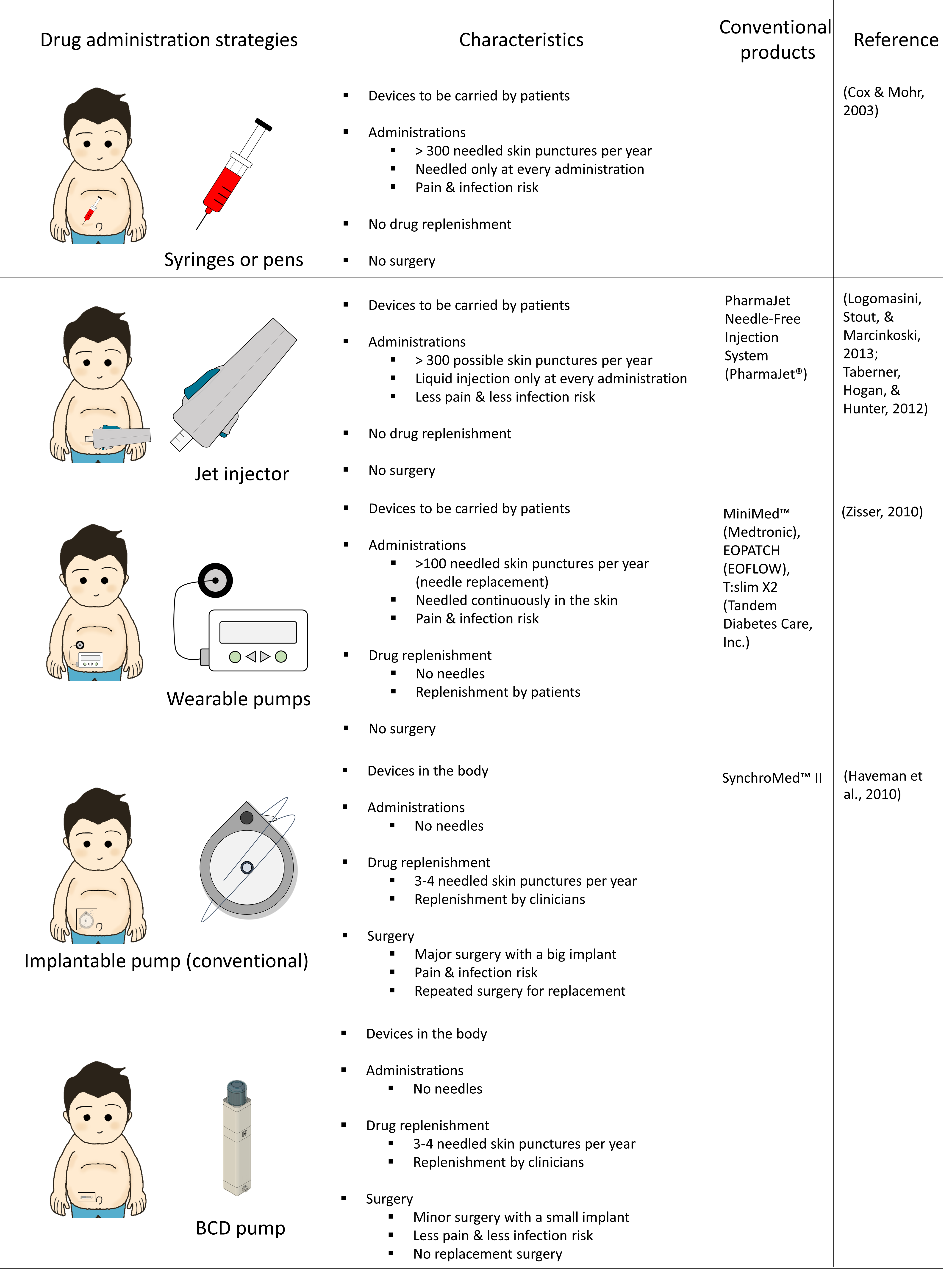


**Supplementary Figure 11. Comparison table of administration strategies for self-injection drugs.**

**Captions for Supplementary Movies**, 1 to 5

**Supplementary Movie 1.** Drug infusion by button clicks with the BCD.

**Supplementary Movie 2.** Reproducible liquid infusion by button clicks with the BCD.

**Supplementary Movie 3.** Profile of force applied on the button of the BCD during click actuation.

**Supplementary Movie 4.** Button click applied while the BCD was still implanted. When the BCD was clicked, the animal without anesthesia did not show any sign of pain or discomfort.

**Supplementary Movie 5.** Drug replenishment while the BCD was still implanted, using a 31 G syringe needle.

**References**

Cobb WS, Burns JM, Kercher KW, Matthews BD, Norton HJ, Heniford BT. Normal intraabdominal pressure in healthy adults. Journal of Surgical Research. 2005;129(2):231-235.

Jeon BS, Shin BH, Huh BK, Kim BH, Kim S-N, Ji HB, et al. Silicone implants capable of the local, controlled delivery of triamcinolone for the prevention of fibrosis with minimized drug side effects. *J Ind Eng Chem*. 2018;63:168-80.

Kim BH, Park M, Park HJ, Lee SH, Choi SY, Park CG, et al. Prolonged, acute suppression of cysteinyl leukotriene to reduce capsular contracture around silicone implants. *Acta Biomater*. 2017;51:209-19.

Cox D, Mohr DC. Managing difficulties with adherence to injectable medications due to blood, injection, and injury phobia and self-injection anxiety. Am. J. Adv. Drug Deliv. 2003;1(3):215-21.

Logomasini MA, Stout RR, Marcinkoski R. Jet injection devices for the needle-free administration of compounds, vaccines, and other agents. International Journal of Pharmaceutical Compounding. 2013;17(4):270-280.

Taberner A, Hogan NC, Hunter IW. Needle-free jet injection using real-time controlled linear Lorentz-force actuators. Medical Engineering & Physics. 2012;34(9):1228-1235.

Zisser HC. The OmniPod Insulin Management System: the latest innovation in insulin pump therapy. Diabetes Therapy. 2010;1(1):10-24.

Haveman JW, Logtenberg SJ, Kleefstra N, Groenier KH, Bilo HJ, Blomme AM. Surgical aspects and complications of continuous intraperitoneal insulin infusion with an implantable pump. Langenbecks Arch Surg. 2010;395(1):65-71.
